# Anchoring Supramolecular Polymers to Human Red Blood Cells by Combining Dynamic Covalent and Non‐Covalent Chemistries

**DOI:** 10.1002/anie.202006381

**Published:** 2020-08-18

**Authors:** Giulia Morgese, Bas F. M. de Waal, Silvia Varela‐Aramburu, Anja R. A. Palmans, Lorenzo Albertazzi, E. W. Meijer

**Affiliations:** ^1^ Laboratory of Macromolecular and Organic Chemistry Institute for Complex Molecular Systems (ICMS) Eindhoven University of Technology 5600MB Eindhoven The Netherlands; ^2^ Department of Biomedical Engineering Institute for Complex Molecular Systems (ICMS) Eindhoven University of Technology 5600MB Eindhoven The Netherlands; ^3^ Institute for Bioengineering of Catalonia (IBEC) The Barcelona Institute of Science and Technology Baldiri Reixac 15–21 08028 Barcelona Spain

**Keywords:** boronic acid, cell/material interactions, multivalency, red blood cells, supramolecular polymers

## Abstract

Understanding cell/material interactions is essential to design functional cell‐responsive materials. While the scientific literature abounds with formulations of biomimetic materials, only a fraction of them focused on mechanisms of the molecular interactions between cells and material. To provide new knowledge on the strategies for materials/cell recognition and binding, supramolecular benzene‐1,3,5‐tricarboxamide copolymers bearing benzoxaborole moieties are anchored on the surface of human erythrocytes via benzoxaborole/sialic‐acid binding. This interaction based on both dynamic covalent and non‐covalent chemistries is visualized in real time by means of total internal reflection fluorescence microscopy. Exploiting this imaging method, we observe that the functional copolymers specifically interact with the cell surface. An optimal fiber affinity towards the cells as a function of benzoxaborole concentration demonstrates the crucial role of multivalency in these cell/material interactions.

## Graphical abstract

**Drawn to the blood**: When benzene‐1,3,5‐tricarboxamide 1D fibers are decorated with benzoxaborole moieties, a dynamic interaction with human red blood cells is induced via the adaptive covalent reaction between boronic acids and cell sialic acids. Total internal reflection fluorescence microscopy is exploited as a powerful tool to allow real‐time imaging of cells/fiber binding, revealing the multivalency of this interaction.

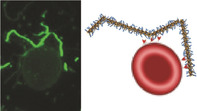

In a superb way, nature exploits multivalent non‐covalent interactions to build complex, dynamic, and functional structures.^1^, ^2^ Mimicking the sophistication of natural systems represents an extremely challenging task for researchers, especially in the design of biomaterials. Utilizing the dynamics and modularity offered by supramolecular and dynamic covalent chemistry,^3^, ^4^, ^5^ it has been possible to introduce fascinating functionalities in artificial systems.^6^, ^7^, ^8^, ^9^, ^10^, ^11^ In particular, supramolecular polymers assembling into 1D fibers in water have been extensively investigated for their similarity with natural fibrillar structures^12^ and even applied as biomaterials.^13^ Peptide amphiphiles^14^ are successfully used for neural regeneration,^15^, ^16^ angiogenesis enhancement,^17^ and atherosclerosis treatment,^18^ while ureido‐pyrimidinone (Upy)‐based materials have been exploited as constituent of elastomeric valve implants for tissue engineering.^19^ Water‐compatible supramolecular benzene‐1,3,5‐tricarboxamide (BTA) polymers are still in their infancy in terms of bioapplications, but they have been comprehensively studied from the fundamental point of view.^20^, ^21^, ^22^ They assemble into 1D fibers via three‐fold hydrogen bonding among the amides and hydrophobic interactions^20^ and they can be made functional through copolymerization.^23^ Carbohydrates,^24^, ^25^, ^26^ DNA,^27^ charges,^28^, ^29^ and peptides^30^ were introduced into the assembled fiber by inducing fiber formation in the presence of selected functionalized monomers. In this way, specific properties were obtained in these supramolecular copolymers. Introducing DNA, for example, paved the way to protein recruitment,^27^ while BTA fibers decorated with charges were able to bind siRNA and allow intracellular delivery in human kidney (HK‐2) cells without showing any cytotoxicity.^28^

In this work, we make a step towards understanding the molecular aspects of cell/material interactions by combining dynamic covalent and non‐covalent chemistries. The well‐known dynamic covalent bond between boronic acid and carbohydrates^31^ is used to induce an interaction between BTA fibers and human red blood cells (hRBCs). In particular, BTA fibers were decorated with different amounts of benzoxaborole moieties at the periphery (BTA‐Ba) and incubated with hRBCs. The latter was chosen for its biological importance and for the presence of a large and varied palette of carbohydrates, including sialic acids, on its membrane.^32^ Benzoxaborole (Ba) was selected as a promising candidate to study the interactions between BTA‐Ba and hRBCs due to its affinity for sialic acid at physiological pH.^33^ This peculiarity is unique for Ba as all known boronic acids dynamically bind carbohydrates in a pH‐dependent fashion.^34^ In order to better understand cell/material interactions, BTA‐Ba/hRBC binding was imaged by means of high‐resolution fluorescence microscopy.

Since accessibility and orientation of the reactive group are essential to enhance binding efficiency, three different BTA‐Ba were synthesized to screen their ability (Scheme 1). BTA‐Ba1 and BTA‐Ba2 each feature only one Ba group either directly connected to the external tetraethylene glycol (BTA‐Ba2) or separated by an additional triethylene glycol (BTA‐Ba1). BTA‐Ba3 instead has three Ba groups. In order to obtain multivalent supramolecular polymers, different percentages of BTA‐Ba monomers (0 %, 10 %, and 25 %) were copolymerized with BTA monomers bearing no Ba (BTA‐3OH) (Figure 1 a,b).

**Figure 1:**
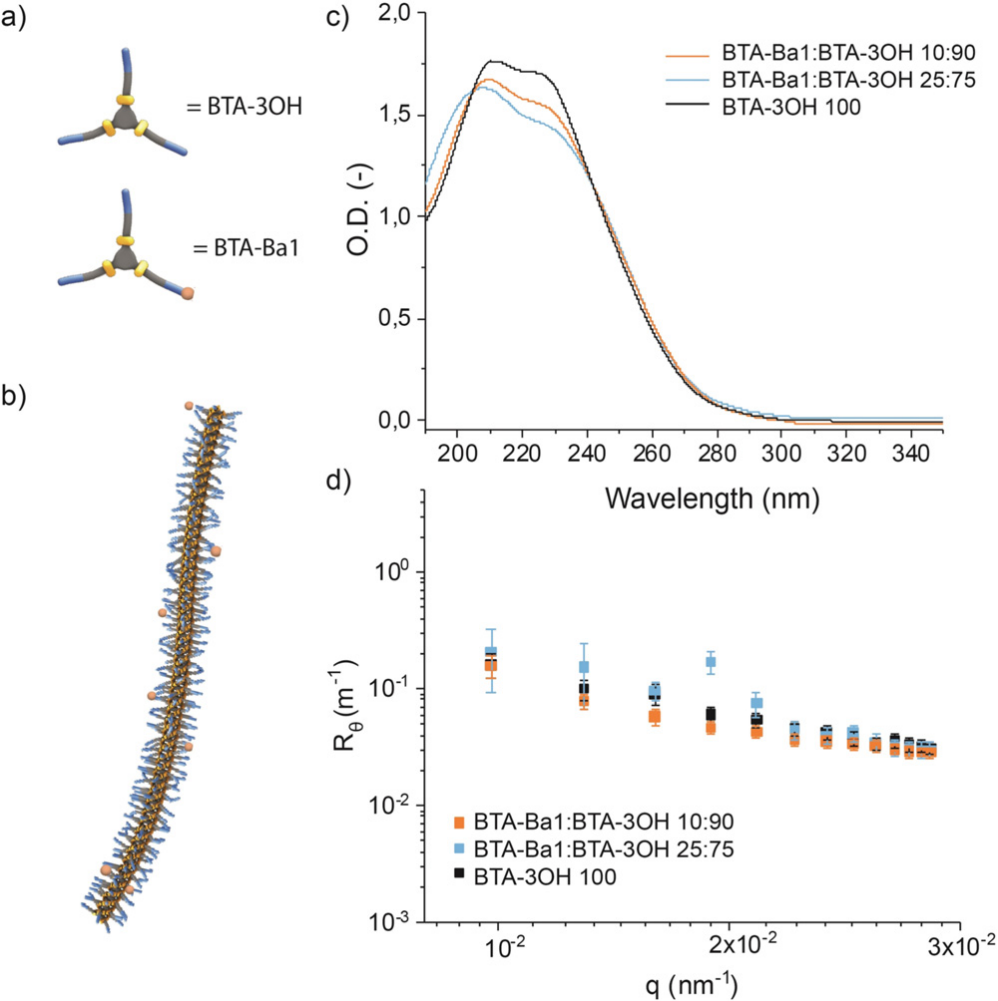
Schematic and chemical representation of BTA‐3OH and BTA‐Ba1 monomers (a) and their assembly into 1D fibers (b). UV/Vis spectra (c) and SLS plots (d) of BTA‐Ba1:BTA‐3OH copolymers in MilliQ water bearing 10 % or 25 % of BTA‐Ba1 compared to those of BTA‐3OH homopolymer.

**Scheme 1:**
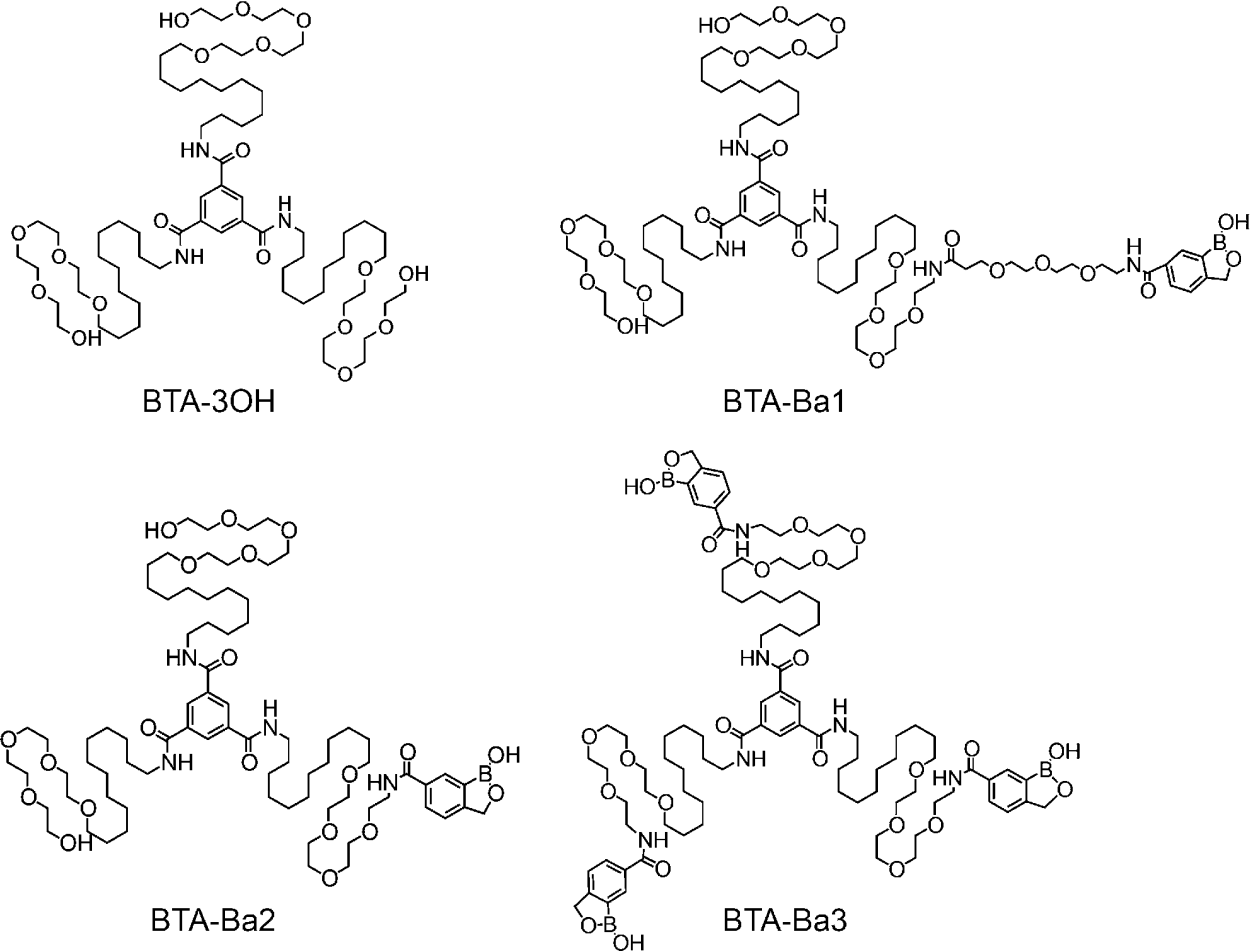
Chemical structures of the four BTA monomers applied in this work.

The formation of 1D fibers upon co‐assembly of the different four monomers (Figure 1 c) was assessed by means of UV/Vis spectroscopy, static light scattering (SLS), cryo‐TEM, and total internal reflection fluorescence (TIRF) microscopy. At all the percentages of BTA‐Ba tested, UV/Vis spectra showed the typical maxima (at 211 nm and 226 nm) of the well‐studied BTA‐3OH (Figures 1 c and Figures S17 a,S19 a in the Supporting Information),^20^ indicating the formation of 1D fibers of the copolymers. SLS further confirmed the assembly into structures with similar morphology and size as BTA‐3OH (Figures 1 d, S17 b, and S19 b). In particular, the best match with BTA‐3OH was achieved by BTA‐Ba1, showing almost overlapping SLS plots. Cryo‐TEM and TIRF microscopy corroborated these results, visualizing fiber‐like conformations for all the different copolymers (Figures 2, S18, and S20). Remarkably, increasing the BTA‐Ba percentage to 25 % induced the coexistence of fibers and smaller aggregates (Figure 2 b), presumably suggesting that 1:4 BTA‐Ba:BTA‐3OH ratio is close to the maximum incorporation of any of the BTA‐Ba into 1D fibers. This assumption was further confirmed by temperature‐induced disassembly studies, monitored by UV/Vis spectroscopy (Figures S21–S26). For all the copolymers, the presence of 25 % of BTA‐Ba resulted in a decrease of the disassembly temperature, indicating a reduced stability of these 1D fibers (Figures S22, S24, and S26). Interestingly, the presence of BTA‐Ba3 as comonomer further enhanced this phenomenon, showing disassembly at lower temperature not only for the 25 % BTA‐Ba3 copolymer but also for the 10 % BTA‐Ba3 (Figures S25 and S26). Based on these UV/Vis and SLS data, we conclude that the copolymers made of BTA‐Ba1 and BTA‐3OH comonomers are the most useful both in terms of morphology and stability. Thus, having only one benzoxaborole per monomer and separating it from the core by an extra spacer showed the least interference with the fiber assembly.

**Figure 2:**
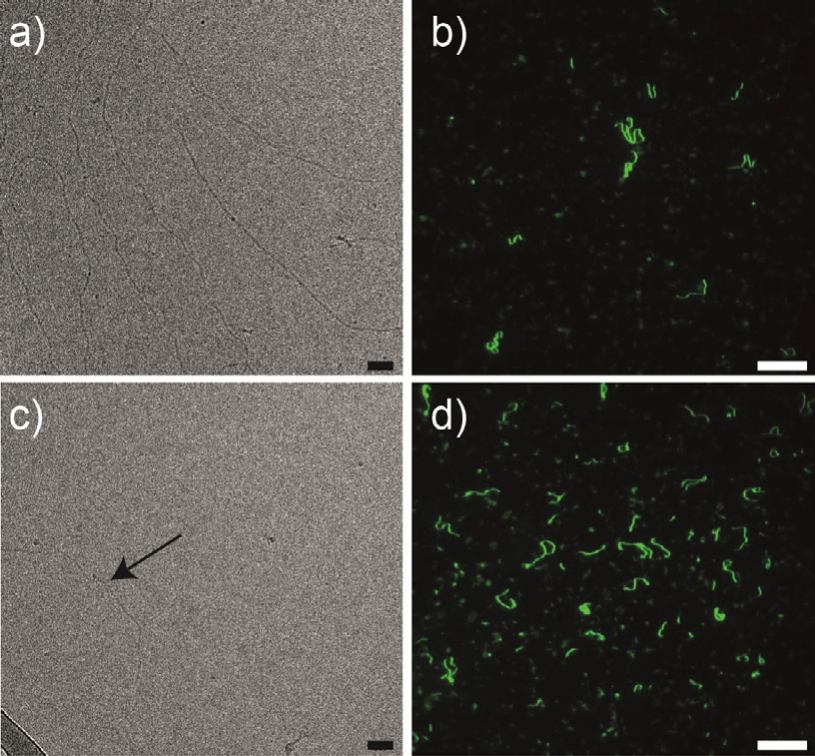
a) Cryo‐TEM images in phosphate buffer saline (PBS) (pH 7.4) of BTA‐Ba1:BTA‐3OH (10:90) and c) of BTA‐Ba1:BTA‐3OH (25:75); scale bar 50 nm. b) TIRF images in PBS (pH 7.4) of BTA‐Ba1:BTA‐3OH (10:90) and d) BTA‐Ba1:BTA‐3OH (25:75); scale bar 10 μm.

The binding between BTA supramolecular copolymers bearing 1 %, 5 % and 10 % BTA‐Ba1 and carbohydrates, that is, sialic acid, was further assessed by means of the Alizarin red S (ARS) assay. ARS is a catechol‐bearing dye, which upon binding to boronic acids becomes fluorescent (*K*_eq_=940 m ^−1^ at pH 7.4).^35^ The addition of carbohydrates that compete with ARS induces fluorescence quenching due to the dissociation of the fluorescent ARS‐boronic acid complex.^36^ All the three copolymers were reacted with ARS in phosphate buffer saline (PBS) to mimic the physiological environment, resulting in intense fluorescence emission proportional to the Ba content (Figures 3 a, S27, and S28).

**Figure 3:**
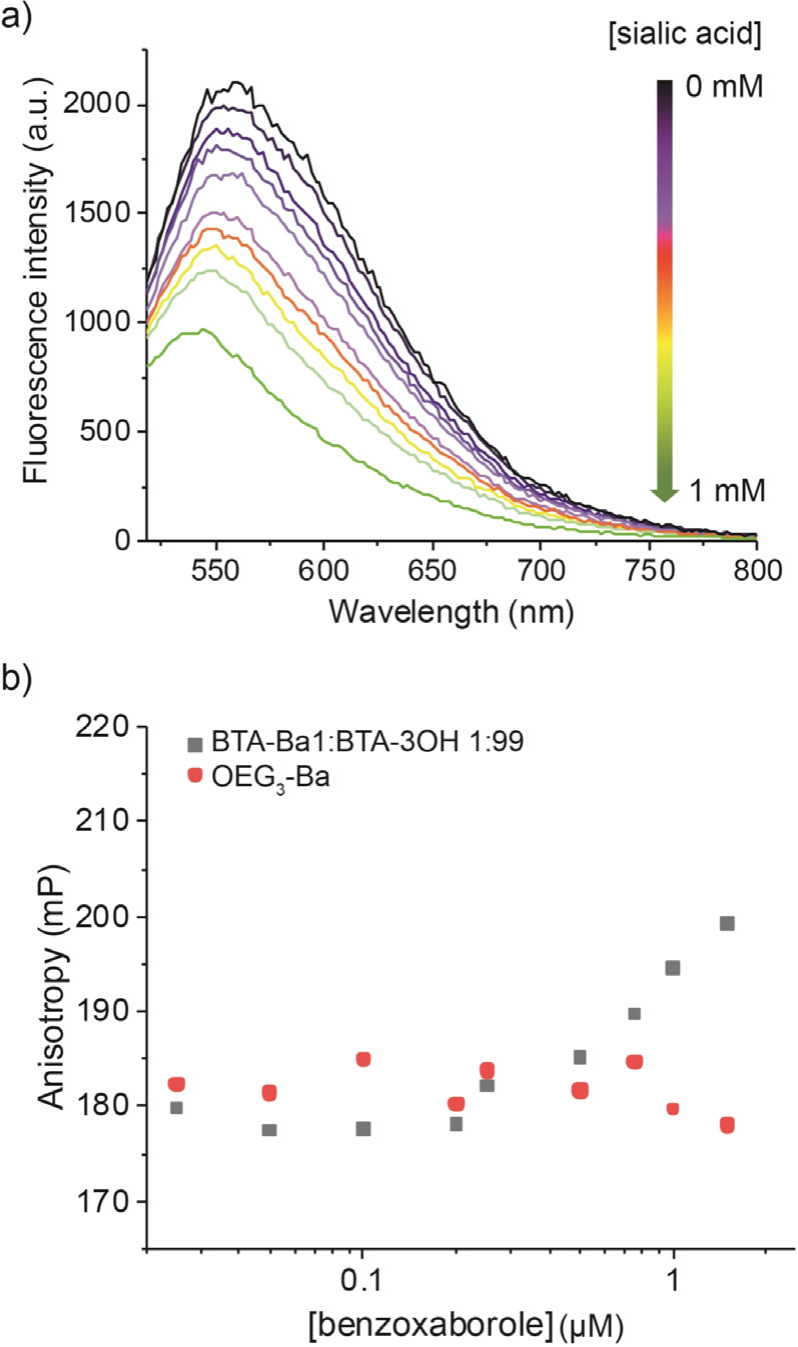
a) Fluorescence emission spectra of a ARS/BTA‐Ba1:BTA‐3OH 1:99 complex upon titration with sialic acid (ARS 20 nm and Ba 5 μm) and b) fluorescence anisotropy of ARS/BTA‐Ba1:BTA‐3OH 1:99 complex compared with that of ARS/OEG_3_‐Ba complex.

Since ARS is highly soluble in water, this first result remarkably showed the accessibility of Ba moieties to the surrounding solvent, explicitly confirming the assumption that they are located at the fiber periphery. The fluorescence was then recorded upon titration with sialic acid and its quenching proved the formation of a complex between BTA‐Ba1:BTA‐3OH fibers and the carbohydrate (Figure 3 a). Interestingly, complete fluorescence quenching was achieved at a 200:1 ratio sialic acid:BTA‐Ba1, in line with the higher *K*_eq_ for ARS to BTA‐Ba1 than sialic acid.

In previous studies, it has been demonstrated that BTA supramolecular fibers are highly dynamic, thus they continuously exchange monomers with the surrounding solvent.^21^, ^22^, ^37^ In order to exclude an interaction between ARS and free BTA‐Ba1 monomers in solution and to further confirm that BTA‐Ba1 monomers are copolymerized with BTA‐3OH, fluorescence anisotropy experiments were performed. Since BTA‐Ba1 monomers are not soluble in water, a hydrophilic surrogate (OEG_3_‐Ba) was synthesized and reacted with ARS. The fluorescence anisotropy of this complex was measured and compared with that generated by ARS/BTA‐Ba1:BTA‐3OH fiber interaction, showing a clear difference between the two situations (Figure 3 b). Binding of ARS with OEG_3_‐Ba resulted in a constant fluorescence anisotropy for all the tested Ba‐concentrations, while the reaction between ARS and the fiber induced an increase in the fluorescence anisotropy at Ba‐concentrations higher than 800 nm. An identical outcome from these two situations would have suggested an interaction between ARS and free BTA‐Ba1 monomers in solution, but the results obtained remarkably indicate that ARS binds to the supramolecular fiber.

Once the functionality of BTA‐Ba1:BTA‐3OH fibers towards sialic acid was determined, fibers bearing different amounts of BTA‐Ba1 were Cy3‐labeled and incubated with hRBCs for 1 h in PBS solution (pH 7.4). Cell–copolymer interactions were then visualized by means of TIRF microscopy (Figure 4 b,c). Interestingly, even at low percentages of incorporated BTA‐Ba1 monomers, that is, 0.1 %, fibers were able to anchor to the cell membrane (Figure S30). Unspecific binding was excluded by performing the incubation of labeled BTA‐3OH fibers with hRBCs, which showed no interaction. (Figure S29) The driving force for the binding of supramolecular polymers on the cell membrane was thus the formation of the dynamic reaction between Ba at the periphery of the fiber and sialic acid at the membrane of the hRBC. By increasing the percentage of BTA‐Ba1, a higher number of cells interacting with fibers was observed (Figures S31–S33), reaching a maximum at 1–5 % of BTA‐Ba1 copolymerized to the inert BTA‐3OH (Figure 4 d).

**Figure 4:**
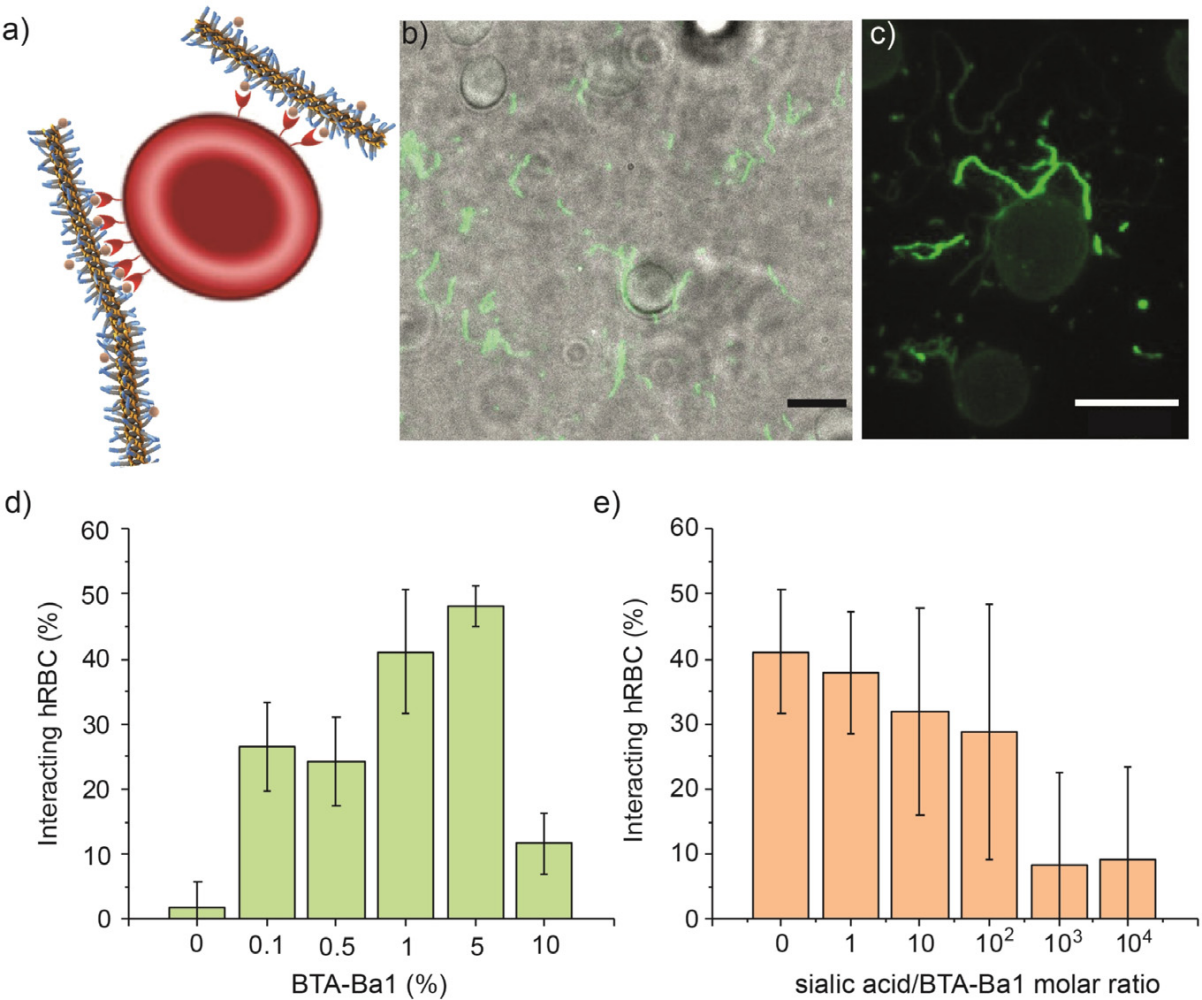
a) Schematic representation of the interaction between BTA‐Ba1 and hRBCs. b) Visualization of the binding of BTA‐Ba1:BTA3OH (1:99) copolymers to the hRBC via superimposition of TIRF and transmission microscopy and c) via TIRF only, scale bars 10 μm. d) Number of hRBCs interacting with BTA‐Ba1:BTA3OH copolymers as a function of the percentage of BTA‐Ba1 comonomers incorporated in the supramolecular fibers and e) competition assay showing the decrease of interaction upon increasing the amount of free sialic acid added to a solution containing BTA‐Ba1:BTA‐3OH copolymers bound to hRBC. Error bars are calculated as standard deviations of the average number of interacting cells.

Remarkably, in many cases the fiber binding is inhomogeneous along the length with bound portions alternating with unbound areas (Figures 4 c, S31 a,e, S33 d, and S35). This corroborates the assumption that the interaction occurs only via the presence of a significant density of Ba/sialic acid binding. In‐situ time lapses (Movies S1,S2 in the Supporting Information) further substantiated this anchoring mechanism, showing BTA‐Ba1:BTA3OH fibers attached only with one extremity onto the hRBC surface and still moving in solution with the other extremity. These results may indicate a clustering of BTA‐Ba1 units within the fiber due to the multivalent interactions with the highly clustered sialic acids at the surface of the hRBCs.^38^ Increasing the percentage of Ba incorporated into the fiber to 10 % surprisingly resulted into less interaction between supramolecular polymers and hRBCs (Figure S34). As mentioned above, increasing the percentage of BTA‐Ba above 10 % resulted in a decrease stability of the fibers, inducing the formation of small aggregates. This destabilization could be enhanced due to binding to the to the clustered sialic acids on hRBCs and the subsequent phase separation of the BTA‐Ba1 from the BTA‐3OH.

Finally, in order to assess whether these cell/fiber interactions were occurring in a multivalent fashion, a competition assay was performed. In particular, BTA‐Ba1:BTA3OH (1:99) fibers were incubated for 1 h in PBS with hRBCs. The fibers detachment from the cell surface upon addition of increasing amounts of free sialic acid in solution was monitored by TIRF microscopy. At low concentrations of free sialic acid, the supramolecular polymers remained attached to the cell membrane (Figures S35,S36), whereas concentrations above 1000‐fold of the total Ba content, almost all the fibers detached from the cells and adsorbed onto the glass surface surrounding the cells (Figures 4 e, S37, and S38). Since the *K*_eq_ of the reaction between Ba and sialic acid is low (160 m ^−1^),^39^ the need for a high amount of free sialic acid to dissociate the Ba/sialic acid complex on the cell membrane is a strong indication of the multivalent binding,^40^ which definitely resulted in overall higher affinity. Compared to the ARS assay, the amount of sialic acid needed to detach the fiber from the cell surface is 5 times higher. This discrepancy is, next to the differences in *K*_eq_, proposed to be correlated to the high density of clustered sialic acid on the hRBCs.

In conclusion, supramolecular polymers were successfully anchored onto the surface of human red blood cells by boronic acid/carbohydrates dynamic covalent chemistry. TIRF microscopy was used for the first time to enable real‐time imaging of these interactions, remarkably demonstrating the role of dynamic multivalency in this binding. The approach presented in this work could be further extended to other cells, providing that they bear carbohydrates on the membrane. Thus, our work opens avenues in the fabrication of biomaterials in which the dynamics and multivalency can be tuned to achieve a more selective and effective interaction with the target cell, with broad implications for drug delivery and regenerative medicine. Furthermore, it represents an enrichment in the field of supramolecular biomaterials, paving the way towards the design of cell/material reciprocity.

## Conflict of interest

The authors declare no conflict of interest.

## Supporting information

As a service to our authors and readers, this journal provides supporting information supplied by the authors. Such materials are peer reviewed and may be re‐organized for online delivery, but are not copy‐edited or typeset. Technical support issues arising from supporting information (other than missing files) should be addressed to the authors.

Supplementary

Supplementary

Supplementary
