# Supplementary material for: Anchoring Supramolecular Polymers to Human Red Blood Cells by Combining Dynamic Covalent and Non‐Covalent Chemistries
Source: Angew Chem Int Ed Engl. 2020 Aug 18;59(39):17229–33. doi: 10.1002/anie.202006381 (PMC7540258; doi:10.1002/anie.202006381)
Supplement: Supplementary file 1 — Supplementary [file ANIE-59-17229-s001.pdf]

Angewandte Chemie
Eine Zeitschrift der Gesellschaft Deutscher Chemiker

Supporting Information

Anchoring Supramolecular Polymers to Human Red Blood Cells by Combining Dynamic Covalent and Non-Covalent Chemistries

Giulia Morgese, Bas F. M. de Waal, Silvia Varela-Aramburu, Anja R. A. Palmans, Lorenzo Albertazzi, and E. W. Meijer**

anie_202006381_sm_miscellaneous_information.pdf
anie_202006381_sm_S1.mov
anie_202006381_sm_S2.mov

Supporting Information

Table of contents

1. Experimental data
2. Supplementary figures and movies
3. References

1. Experimental data

Materials

All the chemicals, except for 1,3-Dihydro-1-hydroxy-2,1-benzoxaborole-6-carboxylic acid (abcr GmbH), sialic acid (abcr GmbH) and t-Boc-N-amido-PEG3-acid (Biochempeg Scientific Inc.), were purchased from Sigma Aldrich. BTA-3OH and Cy3-BTA were synthesized as previously reported.[1,2] Human red blood cells were extracted from human blood (Sanquin Blood Supply, Eindhoven), frozen at -80°C and thawed one day before the experiment.

Synthesis of perfluorophenyl 1-hydroxy-1,3-dihydrobenzo[*c*][1,2]oxaborole-6-carboxylate (PFF-Ba)

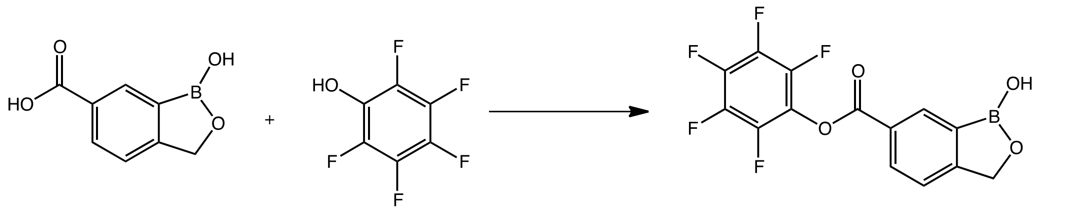

Pentafluorophenol (548 mg; 2.97 mmol) was dissolved in dry dichloromethane (DCM) (11 mL). EDC.HCl (606 mg; 3.16 mmol) was added resulting in a clear solution. 1,3-Dihydro-1-hydroxy-2,1-benzoxaborole-6-carboxylic acid (457 mg; 2.57 mmol) was added resulting in a turbid mixture. After stirring for 1.5h a clear solution was observed. The reaction was left under stirring for other 2.5h and DCM (200 mL) was added. This very turbid mixture was transferred to a separation funnel and shaken with water (100 mL). The water layer was back extracted with DCM (50 mL). The combined DCM fractions were shaken with water (3 x 100 mL) and dried by stirring with sodium sulfate for 20 minutes. After filtration of the salt, the solvent was evaporated, resulting in 0.92 g of crude product. The purification was performed by column chromatography (gradient from 100% EtOAc to 2% v/v MeOH in EtOAc) using a Biotage Isolera One column machine (Biotage 45g ZIP Sphere prepacked silica column). Yield: 0.65 g (73%).

^{1}H NMR (400 MHz, $CDCl_3$) δ = 8.57 (s, 1H, Ar (Ba)), 8.28 (d, *J*=8.1 Hz, 1.7, 1H, Ar (Ba)), 7.52 (d, *J*=8.1 Hz, 1H, Ar (Ba)), 5.20 (s, 2H, CH_2-O).
^{13}C NMR (100 MHz, DMSO) δ 162.89, 161.66, 133.59, 132.88, 125.19, 123.23, 70.68, 49.06.

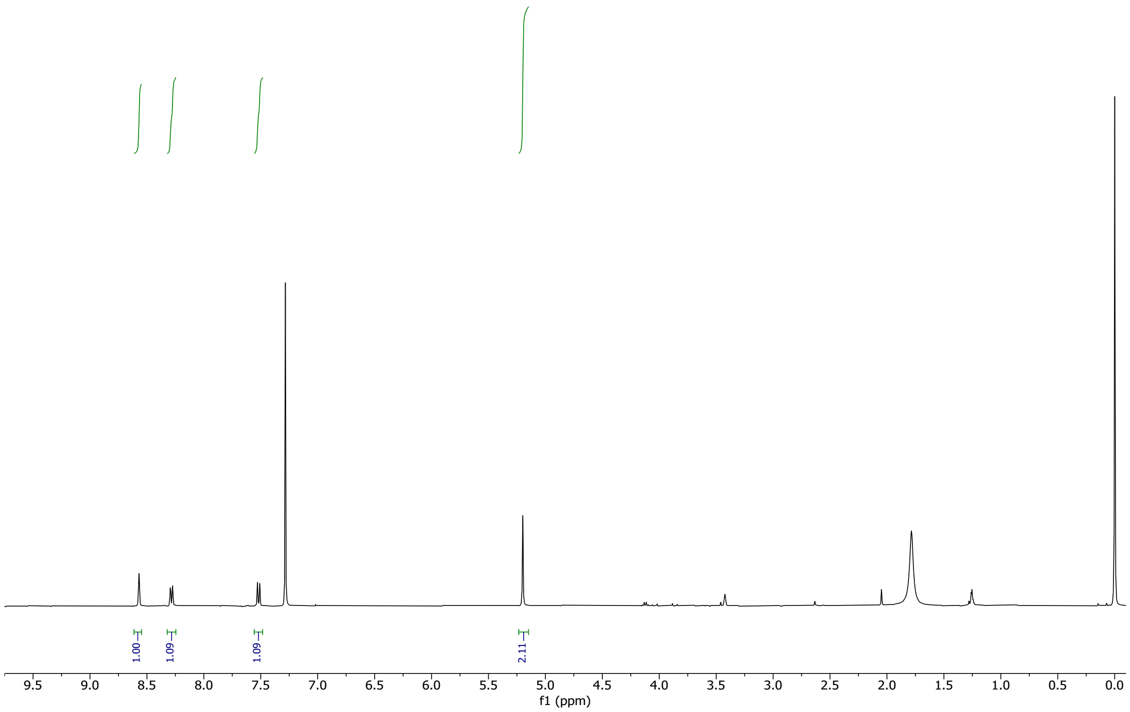

Figure S1. ^{1}H-NMR spectrum of PFF-Ba in $CDCl_3$ with trace amount of CD_3OD added for solubility reasons.

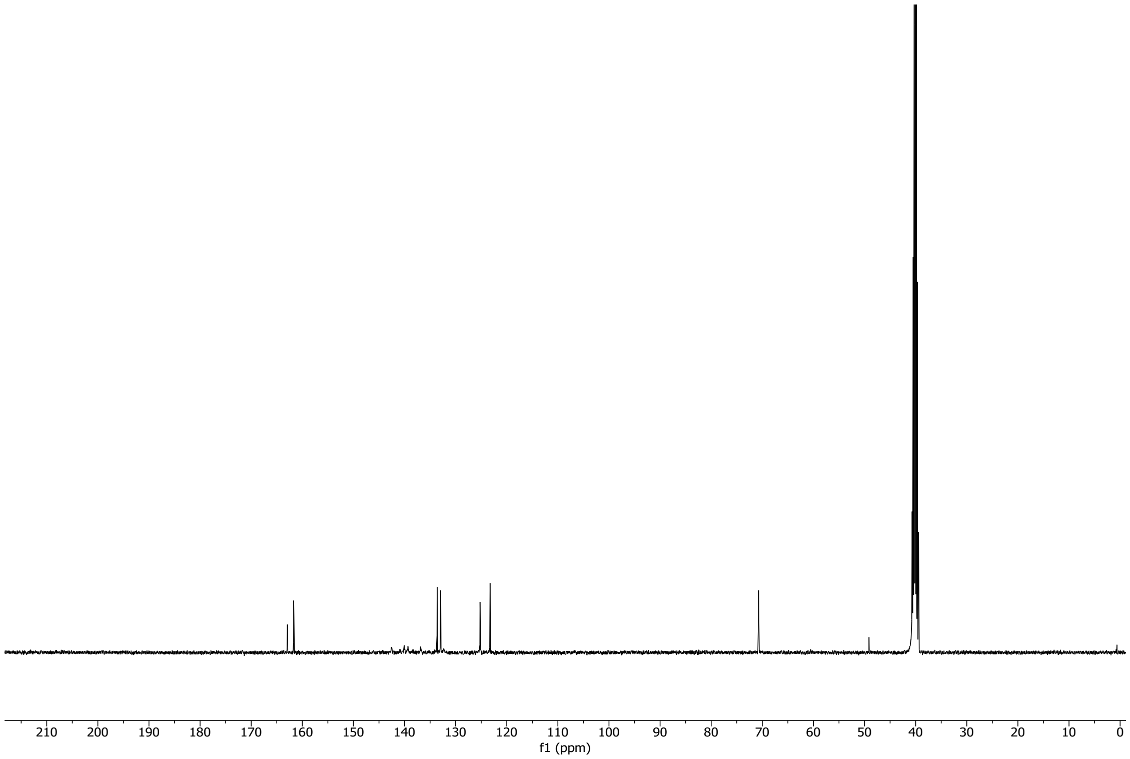

Figure S2. ^{13}C-NMR spectrum of PFF-Ba in DMSO-d_6.

Synthesis of 2,5-dioxopyrrolidin-1-yl 2,2-dimethyl-4-oxo-3,8,11,14-tetraoxa-5-azaheptadecan-17-oate (t-Boc-N-amido-PEG3-NHS ester)

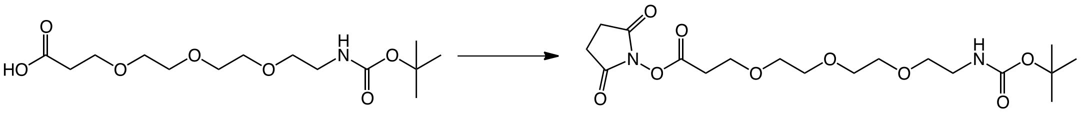

2,2-dimethyl-4-oxo-3,8,11,14-tetraoxa-5-azaheptadecan-17-oic acid (t-Boc-N-amido-PEG3-acid) (926 mg; 2.88 mmol) was dissolved in dry DCM (10 mL) and N-Hydroxysuccinimide (420 mg; 3.65 mmol) was added, followed by EDC.HCl (720 mg; 3.76 mmol). After stirring for 25h, DCM (250 mL) was further added and the solution was transferred to a separation funnel. The organic phase was extracted with water (3 x 130 mL), and brine (250 mL). After drying on magnesium sulfate, 1.20 g of pure product was obtained (yield: 99%)

^{1}H NMR (400 MHz, $CDCl_3$) δ = 3.86 (t, *J*=6.4 Hz, 2H, C=ONHCH$_2$C$\underline{H_2}$O), 3.69 – 3.50 (m, 10H, OC$\underline{H_2}$C$\underline{H_2}$O), 3.36 – 3.26 (m, 2H, C=ONHC$\underline{H_2}$CH$_2$O), 2.91 (t, *J*=6.4 Hz, 2H, OC=OC$\underline{H_2}$), 2.84 (s, 4H, , CH$_2$ NHS-ester), 1.44 (s, 9H, CH$_3$).
^{13}C NMR (100 MHz, $CDCl_3$) δ 168.92, 166.73, 156.01, 79.16, 77.34, 77.02, 76.70, 70.79-70.21, 65.75, 40.38, 32.18, 28.44, 25.59.
MS (MALDI-TOF) calcd. for $C_{18}H_{30}2_{5}O_{9}$: 441.18 [M + Na]$^+$, found 441.22; calcd. 457.16 [M + K]$^+$, found 457.21

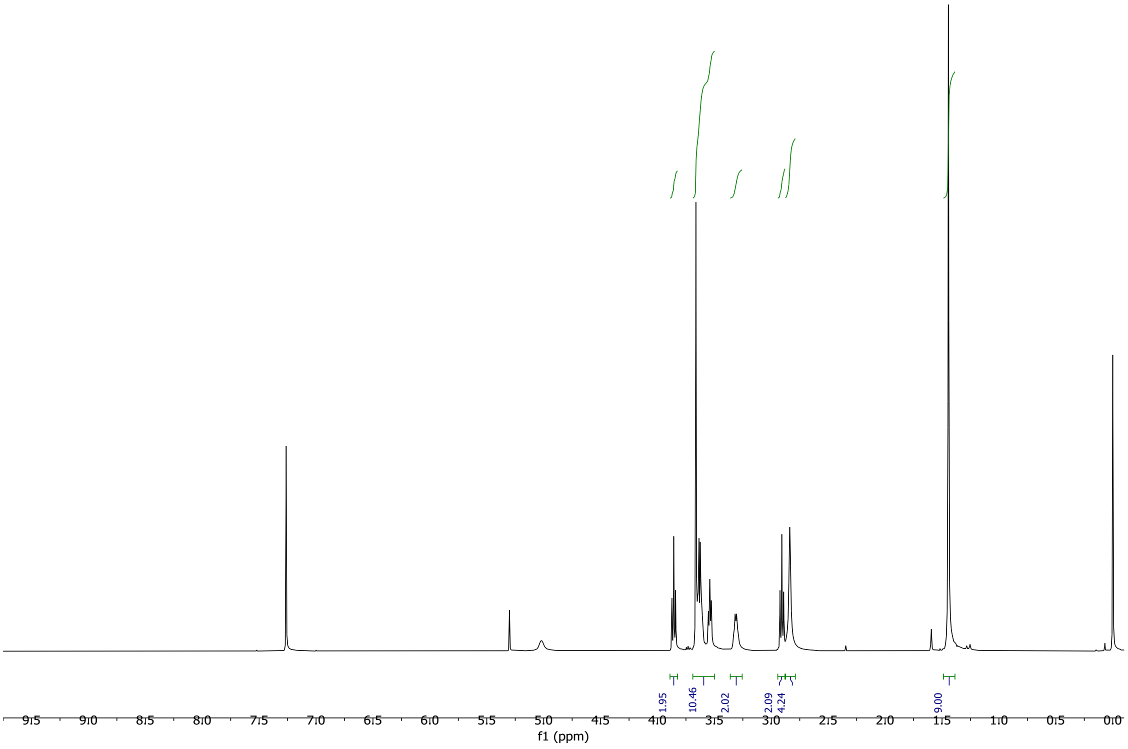

Figure S3. ^{1}H-NMR spectrum of t-Boc-N-amido-PEG3-NHS ester in $CDCl_3$.

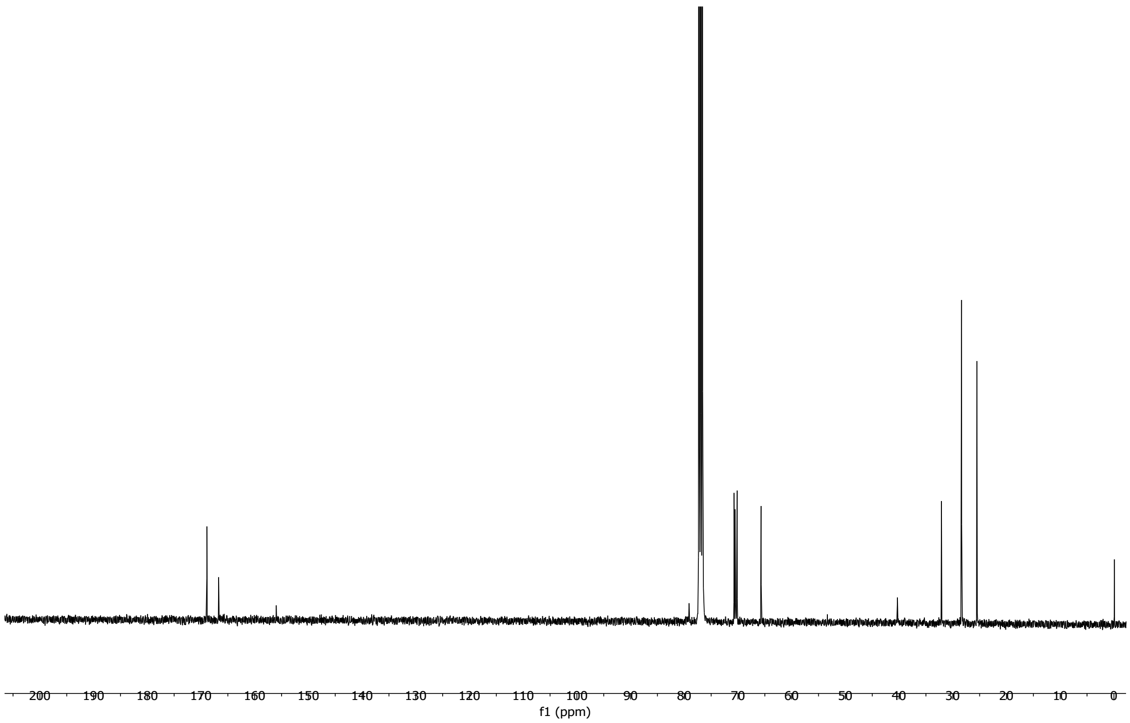

Figure S4. ^{13}C-NMR spectrum of t-Boc-N-amido-PEG3-NHS ester in $CDCl_3$.

Synthesis of *N*[1]-(1-(1-hydroxy-1,3-dihydrobenzo[*c*][1,2]oxaborol-6-yl)-1,14-dioxo-5,8,11,18,21,24,27-heptaoxa-2,15-diazanonatriacontan-39-yl)-*N*[3],*N*[5]-bis(1-hydroxy-3,6,9,12-tetraoxatetracosan-24-yl)benzene-1,3,5-tricarboxamide (BTA-Ba1)

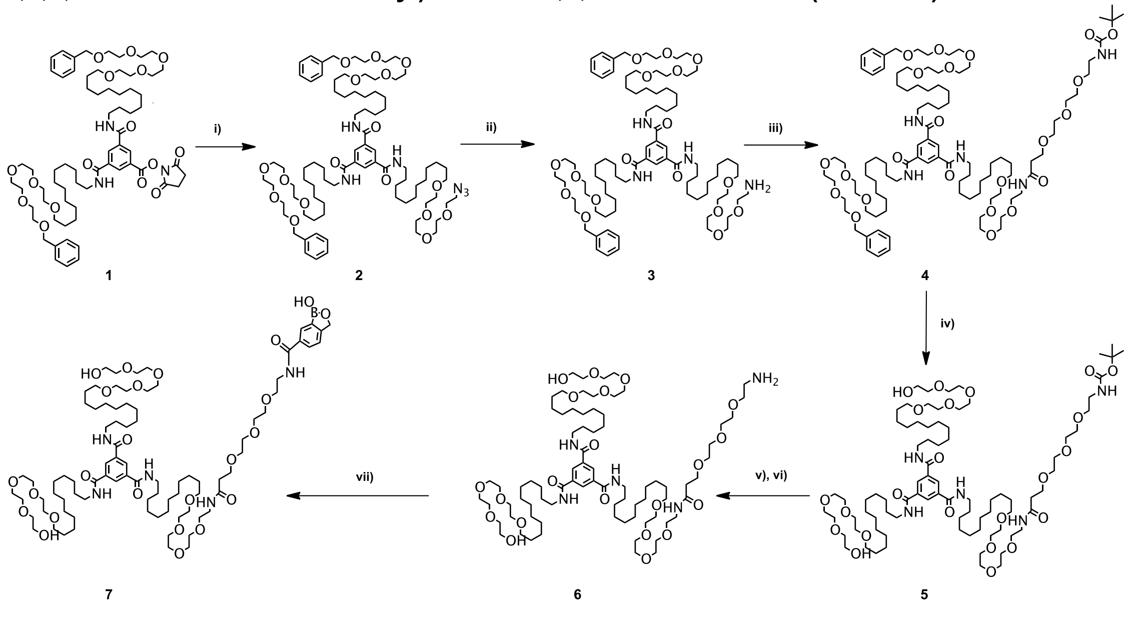

Scheme S1. Synthesis of BTA-Ba1. i) 1-azido-3,6,9,12-tetraoxatetracosan-24-amine[2]/TEA, ii) triphenyl phosphine/ H_2O, iii) t-Boc-N-amido-PEG3-NHS ester/TEA, iv) H_2, Pd/C, v) TFA, vi) $LiOH.H_2O$, vii) PFF-Ba/TEA.

- ***Synthesis of N[1]-(1-azido-3,6,9,12-tetraoxatetracosan-24-yl)-N[3],N[5]-bis(1-phenyl-2,5,8,11,14-pentaoxahexacosan-26-yl)benzene-1,3,5-tricarboxamide (2)***

2,5-dioxopyrrolidin-1-yl-3,5-bis((1-phenyl-2,5,8,11,14-pentaoxahexacosan-26-yl) carbamoyl)benzoate (BTA-NHS) (**1**)[3] (200 mg; 0.16 mmol) was dissolved in 5 mL DCM and triethylamine (0.10 ml; 0.73 mmol) was added. To this mixture a solution of 1-azido-3,6,9,12-tetraoxatetracosan-24-amine[2] (77 mg; 0.19 mmol) in DCM (2 mL) was

further added. After 20 h stirring at room temperature, DCM (70 mL) was added and the reaction mixture was transferred into a separation funnel. 1M $KHSO_4$ (aq, 20 mL) was added and the mixture shaken. The water layer was again extracted with DCM (20 mL). The combined organic phases were washed with 1M $KHSO_4$ (aq, 20 mL) and this water phase was further washed with chloroform (20 mL). The combined organic phases were further shaken with saturated $NaHCO_3$ (aq, 20 mL), followed by brine (25 mL) and then dried on magnesium sulfate. The purification was performed by column chromatography (gradient from $CHCl_3$ to $CHCl_3$:MeOH 93:7 v/v) on a Biotage Isolera One column machine (Büchi 4g prepacked silica column) and the product **2** was obtained. Yield: 206 mg (84%).

^1H NMR (400 MHz, $CDCl_3$) δ = 8.36 (s, 3H, Ar), 7.36 – 7.28 (m, 10H, Ar), 6.60 – 6.52 (m, 3H, $CH_2N\underline{H}C{=}O$), 4.55 (s, 4H, Ar-$C\underline{H}_2$-O), 3.68 – 3.54 (m, 46H, O-$(C\underline{H}_2)_2$-O), 3.48 – 3.40 (m, 12H, $CH_2C\underline{H}_2NHC{=}O$, $CH_2CH_2C\underline{H}_2O$), 3.38 (t, J = 5.0 Hz, 2H, $C\underline{H}_2$-N_3), 1.56 – 1.17 (m, 60H, aliphatic).
^{13}C NMR (100 MHz, $CDCl_3$) δ = 165.67, 138.22, 135.27, 128.36-127.61, 77.34, 77.03, 76.71, 73.26, 71.56-69.44, 50.70, 40.38, 29.61, 29.56, 29.51, 29.46, 29.42, 29.23, 26.95, 26.07.
MS (MALDI-TOF) calcd. for $C_{83}H_{140}6_5O_{17}$ 1516.01 $[M + Na]^+$, found 1516.05.

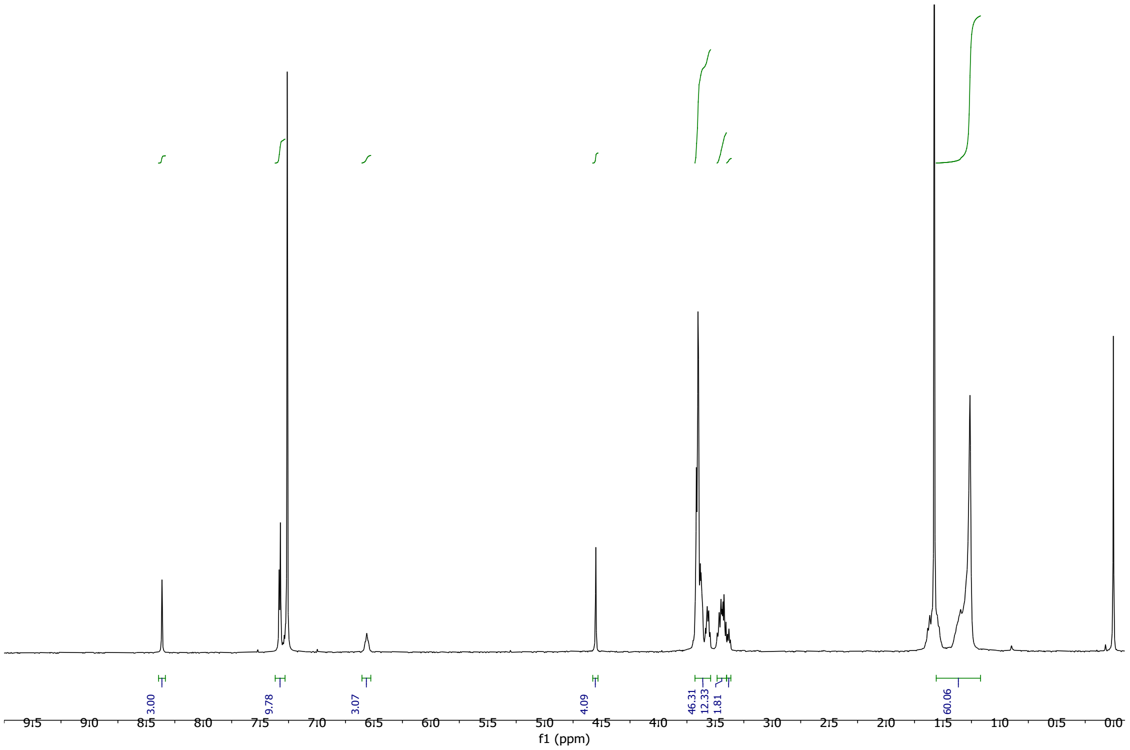

Figure S5. ^1H-NMR spectrum of **2** in $CDCl_3$.

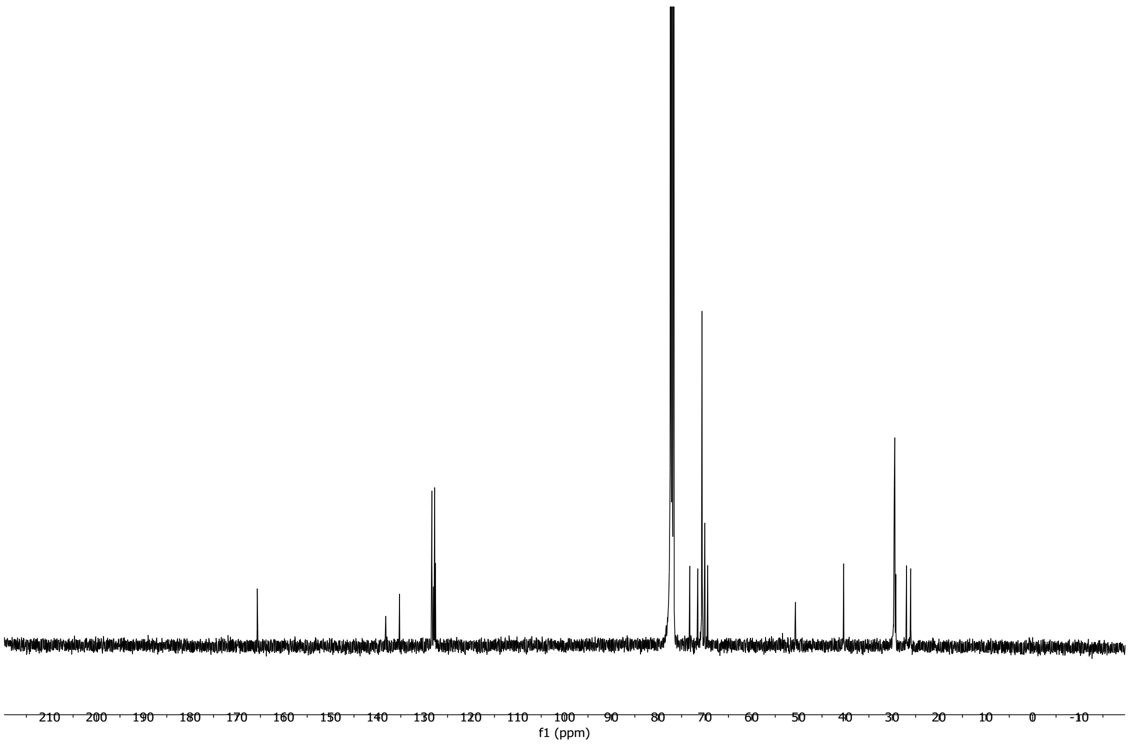

Figure S5. ^{13}C-NMR spectrum of **2** in $CDCl_3$.

- ***Synthesis of N^{1}-(1-amino-3,6,9,12-tetraoxatetracosan-24-yl)-N^{3},N^{5}-bis(1-phenyl-2,5,8,11,14-pentaoxahexacosan-26-yl)benzene-1,3,5-tricarboxamide (3)***

To a stirred solution of **2** (300 mg; 0.20 mmol) in THF, water (6 mL) was added. Triphenyl phosphine (112 mg) was further added, obtaining a clear solution, which was stirred for 28 h at 50 °C. After this time, the solution was concentrated on a rotary evaporator. The purification was performed by column chromatography on a Biotage Isolera One column machine (10 g Biotage KP-SIL prepacked silica column), firstly eluting the phosphorus containing products with EtOAc 100% and then eluting the product (**3**) with $CHCl_3$:MeOH:isopropylamine 90:8:2 v/v/v. Yield: 270 mg (92 %). The successful reduction of the azide to amine was confirmed by ^{1}H-NMR.

^{1}H NMR (400 MHz, $CDCl_3$) δ = 8.37 (s, 3H, Ar), 7.36 – 7.27 (m, 10H, Ar), 6.68 – 6.59 (m, 3H, $CH_2N\underline{H}C{=}O$), 4.55 (s, 4H, Ar-$C\underline{H}_2$-O), 3.67 – 3.54 (m, 46H, O-$(C\underline{H}_2)_2$-O), 3.50 – 3.42 (m, 12H, $CH_2C\underline{H}_2NHC{=}O$, $CH_2CH_2C\underline{H}_2O$), 2.85 (t, *J*=5.3 Hz, 2H, $C\underline{H}_2$-NH_2), 1.27 (d, *J*=8.8 Hz, 60H, aliphatic).

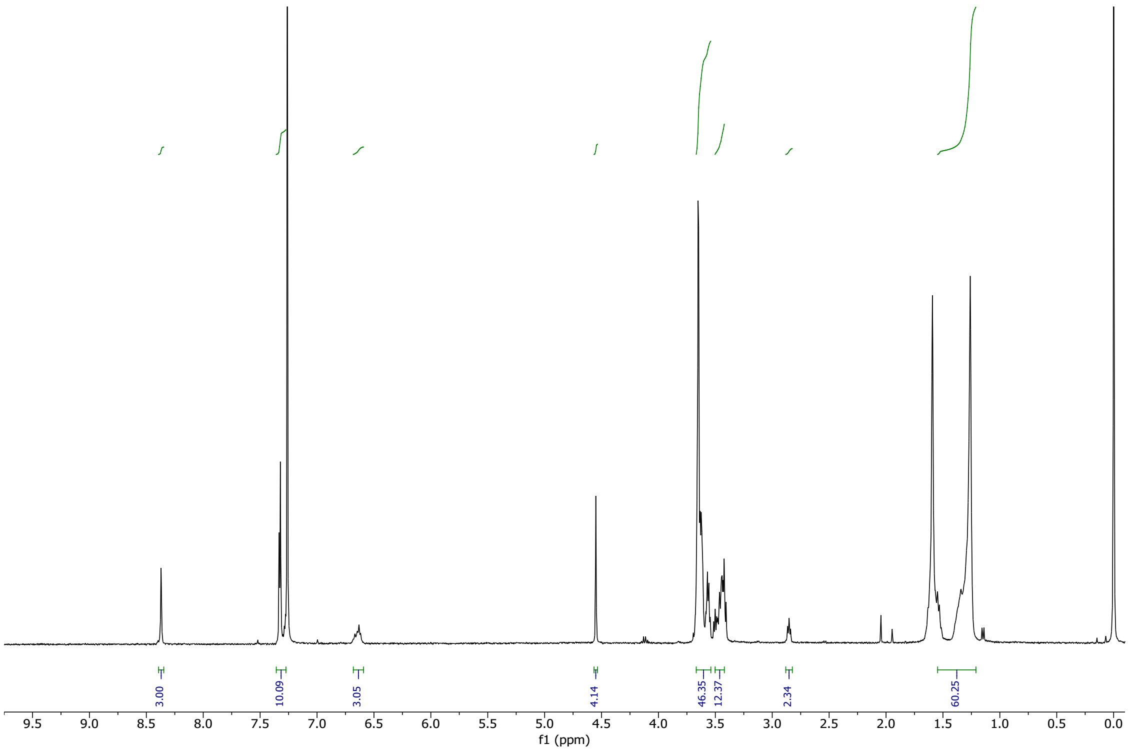

Figure S6. ^{1}H-NMR spectrum of **3** in $CDCl_3$.

- ***Synthesis of tert-butyl (1-(3,5-bis((1-phenyl-2,5,8,11,14-pentaoxahexacosan-26-yl)carbamoyl)phenyl)-1,28-dioxo-15,18,21,24,31,34,37-heptaoxa-2,27-diazanonatriacontan-39-yl)carbamate (4)***

A solution of t-Boc-N-amido-PEG3-NHS ester (82 mg; 0.196 mmol) in DCM (1 mL) was added to a solution of **3** (270 mg; 0.18 mmol in 10 mL DCM) and triethylamine (0.1 mL). After 22h, *Argopore*-NH_2-LL resin (136 mg) was added to remove the unreacted active ester. After other 15h, the reaction mixture was filtered. The resin was washed with DCM (20 mL) and the filtrate was transferred to a separation funnel and shaken with 1M $KHSO_4$ (aq, 20 mL). The water layer was extracted with DCM (20 mL). The combined organic fractions were washed with 1M $KHSO_4$ (aq, 20 mL) and saturated sodium bicarbonate (aq, 20 mL), followed by brine (20 mL). The purification was performed by column chromatography (chloroform:methanol 10:1 v/v) on a Biotage Isolera One column machine (10 g ZIP sphere silica column) and 237 mg (yield 72%) of **4** were obtained.

^{1}H NMR (400 MHz, $CDCl_3$) δ = 8.37 (s, 3H, Ar), 7.37 – 7.26 (m, 10H, Ar), 6.75 – 6.60 (m, 3H, $CH_2N\underline{H}C$=O), 4.55 (s, 4H, , Ar-$C\underline{H}_2$-O), 3.74 (t, *J*=6.0 Hz, 2H, C=ONHCH_2C$\underline{H}_2$O), 3.68 – 3.50 (m, 56H, O-(C$\underline{H}_2$)$_2$-O), 3.48 – 3.38 (m, 14H, , $CH_2C\underline{H}_2$NHC=O, $CH_2CH_2C\underline{H}_2$O), 3.33 – 3.27 (m, 2H, $C\underline{H}_2$NHC=O), 2.47 (t, *J*=6.0 Hz, 2H, NHC=OC$\underline{H}_2$), 1.64 – 1.51 (m, 12H, $CH_2C\underline{H}_2CH_2$O, $C\underline{H}_2CH_2$NHC=O), 1.44 (s, 9H, CH_3), 1.38 – 1.20 (m, 48H, aliphatic).
MS (MALDI-TOF) calcd. for $C_{97}H_{167}N_5O_{23}$ 1794.19 [M + Na]$^+$, found 1794.23.

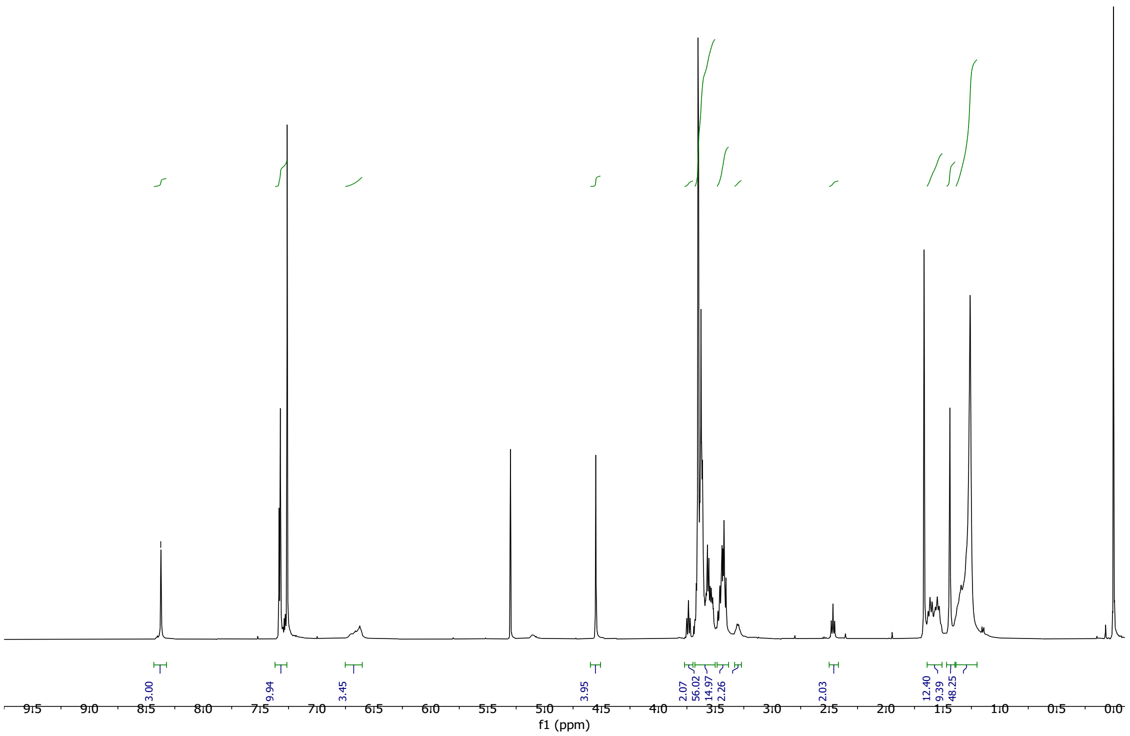

Figure S7. ^{1}H-NMR spectrum of **4** in $CDCl_3$.

- ***Synthesis of tert-butyl (1-(3,5-bis((1-hydroxy-3,6,9,12-tetraoxatetracosan-24-yl)carbamoyl)phenyl)-1,28-dioxo-15,18,21,24,31,34,37-heptaoxa-2,27-diazanonatriacontan-39-yl)carbamate (5)***

4 (237 mg; 0.14 mmol) was dissolved in absolute methanol (30 mL) and stirred. Nitrogen gas was bubbled through for 10 minutes and 3 drops of acetic acid were added. Pd/C 10% (20 mg; Merck) was added, and a hydrogen gas filled balloon was attached to the flask. After 18h, the mixture was filtered and the filter content rinsed with methanol (25 mL). Evaporation of the solvent yielded the product **5**. Yield: 211 mg (95 %).

^{1}H NMR (400 MHz, $CDCl_3$) δ = 8.40 (s, 3H, Ar), 6.76 (m, 3H, $CH_2N\underline{H}C{=}O$), 3.77 – 3.68 (m, 6H, $C{=}ONHCH_2C\underline{H_2}O$, $C\underline{H_2}OH$), 3.68 – 3.48 (m, 52H, $O\text{-}(C\underline{H_2})_2\text{-}O$), 3.49 – 3.39 (m, 14H, $CH_2C\underline{H_2}NHC{=}O$, $CH_2CH_2C\underline{H_2}O$), 3.32 – 3.30 (m, 2H, $C\underline{H_2}NHC{=}O$), 2.47 (t, *J*=6.1 Hz, 2H, $NHC{=}OC\underline{H_2}$), 1.64 – 1.51 (m, 12H, $CH_2C\underline{H_2}CH_2O$, $C\underline{H_2}CH_2NHC{=}O$), 1.44 (s, 9H, CH_3), 1.38 – 1.20 (m, 48H, aliphatic).
MS (MALDI-TOF) calcd. for $C_{83}H_{155}N_5O_{23}$ 1613.09 [M + Na]$^+$, found 1613.11.

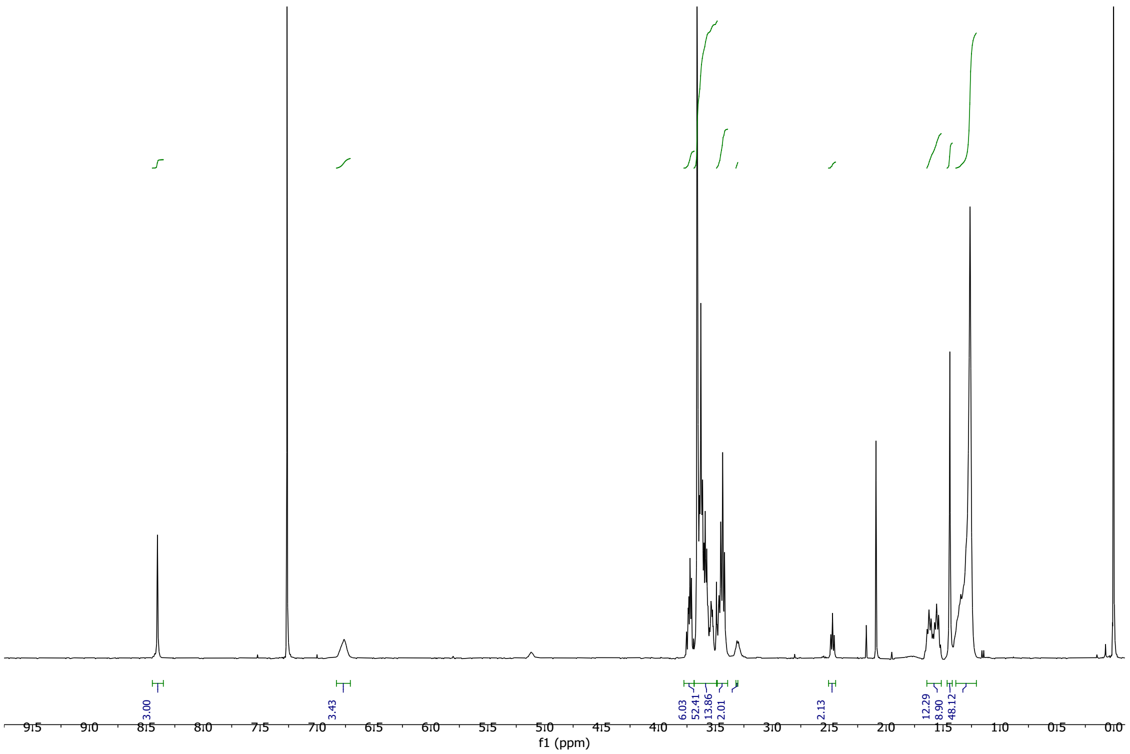

Figure S8. ^{1}H-NMR spectrum of **5** in $CDCl_3$.

- ***Synthesis of N[1]-(1-amino-12-oxo-3,6,9,16,19,22,25-heptaoxa-13-azaheptatriacontan-37-yl)-N[3],N[5]-bis(1-hydroxy-3,6,9,12-tetraoxatetracosan-24-yl)benzene-1,3,5-tricarboxamide (6)***

5 (205 mg; 0.13 mmol) was dissolved in dry DCM (10 mL) and TFA (0.50 mL) was added. After 17h of stirring, DCM (50 mL) was added and the solution was transferred in a separation funnel. Water (6 mL) and 1N NaOH (aq; 10 mL) were added and the mixture was shaken. The water layer was extracted with DCM (20 mL). The organic fractions were combined and washed with brine (20 mL). After Na_2SO_4 drying, 235 mg of crude product was obtained. Since TFA reacts also with the alcohols producing TFA esters, those need to be hydrolysed. Thus, the crude product was dissolved in methanol (6 mL) and water (0.5 mL). Then $LiOH.H_2O$ (40 mg) was added. After 16 hours stirring at room temperature, the mixture was concentrated. Then chloroform (70 mL) and water (10 mL) were added and the mixture transferred in a separation funnel. Some sodium chloride was added to increase phase separation. After concentration of the organic layer, the crude was purified by column chromatography on a Biotage Isolera One column machine. First impurities were eluted by using $CHCl_3$:MeOH 90:10 v/v. The product **6** was obtained by eluting with 90:8:2 $CHCl_3$:MeOH: isopropylamine v/v/v. Yield: 125 mg (63 %). The successful BOC deprotection was confirmed by ^{1}H-NMR.

^{1}H NMR (400 MHz, $CDCl_3$) δ = 8.41 (s, 3H, Ar), 3.75 – 3.70 (m, 6H, $C{=}ONHCH_2C\underline{H_2}O$, $C\underline{H_2}OH$), 3.69 – 3.55 (m, 52H, O-$(C\underline{H_2})_2$-O), 3.47 – 3.38 (m, 14H, $CH_2C\underline{H_2}NHC{=}O$, $CH_2CH_2C\underline{H_2}O$), 2.91 (t, J=7.8 Hz, 2H, $C\underline{H_2}NH_2$), 2.47 (t, J=5.8 Hz, 2H, $NHC{=}OC\underline{H_2}$), 1.49 – 1.20 (m, 60H, aliphatic).

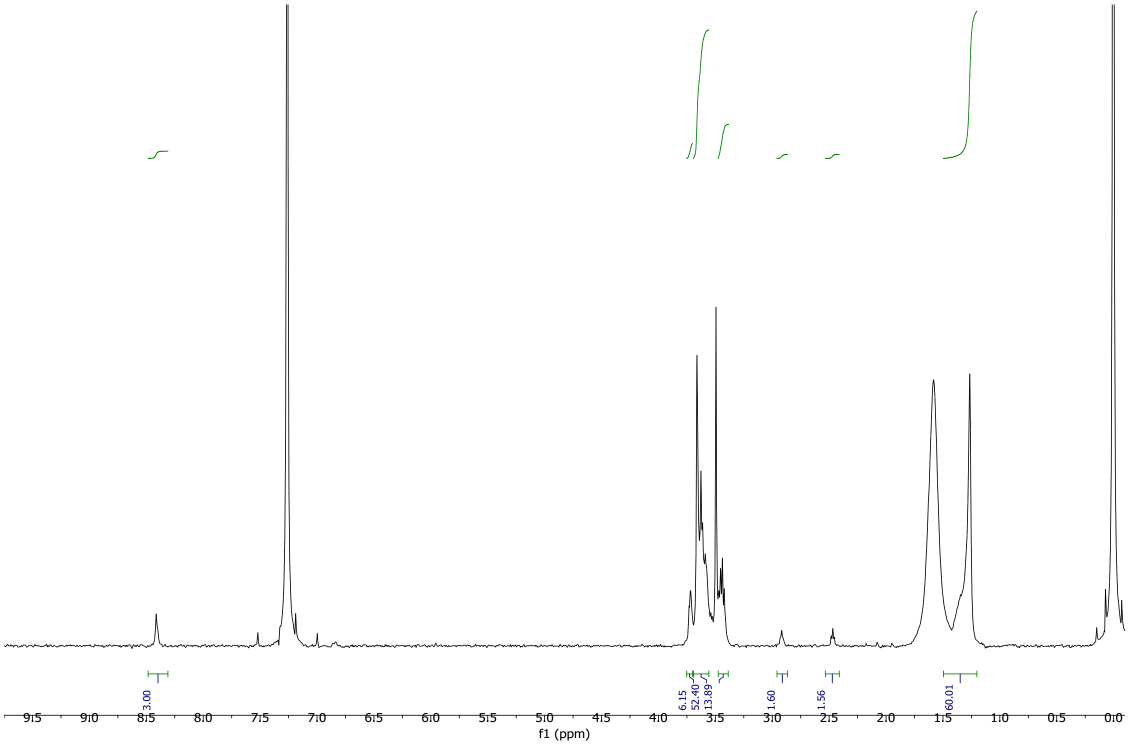

Figure S9. ^{1}H-NMR spectrum of **6** in $CDCl_3$.

- ***Synthesis of N^1-(1-(1-hydroxy-1,3-dihydrobenzo[c][1,2]oxaborol-6-yl)-1,14-dioxo-5,8,11,18,21,24,27-heptaoxa-2,15-diazanonatriacontan-39-yl)-N^3,N^5-bis(1-hydroxy-3,6,9,12-tetraoxatetracosan-24-yl)benzene-1,3,5-tricarboxamide (7)***

6 (77.3 mg; 0.05 mmol), 2-methoxyethanol (20 mg; 0.26 mmol) and triethylamine (20 mg; 0.20 mmol) were dissolved in $CHCl_3$ (1 mL) and stirred under Argon. PFF-Ba (18.3 mg; 0.05 mmol) was added, resulting in a clear solution. After 19 h, a ^{1}H-NMR sample showed complete reaction and the presence of some unreacted PFF-Ba. In order to remove this PFF-Ba excess, *Argopore*-NH_2-LL resin (55 mg of resin with a loading of 0.28 mmol NH_2 per gram) was added and the mixture was stirred for 16h. The mixture was then filtered, the filtrated resin was washed with $CHCl_3$ (40 mL) and the organic solution concentrated. The solid was redissolved into $CHCl_3$ (50 mL), washed with saturated aqueous ammonium chloride (3 x 10 mL), followed by brine (20 mL) and resulting in 66.6 mg of crude product. The purification was performed by column chromatography, firstly eluting impurities with EtOAc:MeOH 90:10 v/v and then eluting the wanted product (**7**) with $CHCl_3$:MeOH 80:20 v/v. Yield: 52 mg (61%).

^{1}H NMR (400 MHz, DMSO) δ = 9.29 (s, 1H, B-O$\underline{H}$), 8.63 (t, *J*=5.6 Hz, 3H, $CH_2N\underline{H}C=O$), 8.51 (t, *J*=5.6 Hz, 1H, Ar (Ba)), 8.36 (s, 3H, Ar), 8.22 (s, 1H, $CH_2N\underline{H}C=OCH_2$), 7.93 (d, *J*=8.0 Hz, 1H, Ar (Ba)), 7.47 (d, *J*=8.0 Hz, 1H, Ar (Ba),), 5.03 (s, 2H, $C\underline{H_2}$-O-B), 4.56 (s, 2H, OH), 3.59 – 3.53 (m, 6H, $C=ONHCH_2C\underline{H_2}O$, $C\underline{H_2}OH$), 3.51 – 3.40 (m, 52H, O-$(C\underline{H_2})_2$-O), 3.37 – 3.31 (m, 14H, $CH_2C\underline{H_2}NHC=O$, $CH_2CH_2C\underline{H_2}O$), 3.21 – 3.16 (m, 2H, $C\underline{H_2}NHC=O$), 2.30 (t, *J*=6.5 Hz, 2H, $CH_2NHC=OC\underline{H_2}$), 1.55 – 1.39 (m, 12H, $CH_2C\underline{H_2}CH_2O$, $C\underline{H_2}CH_2NHC=O$), 1.31 – 1.22 (m, 48H, aliphatic).
^{13}C NMR (100 MHz, $CDCl_3$) δ = 165.95, 135.25, 128.24, 77.34, 77.03, 76.71, 72.58, 71.56, 70.60-69.68, 67.21, 61.69, 40.39, 39.97, 39.39, 36.74, 29.51-29.21, 26.94, 26.02.
MS (MALDI-TOF) calcd. for $C_{86}H_{152}BN_5O_{24}$: 1673.08 $[M + Na]^+$, found 1673.11

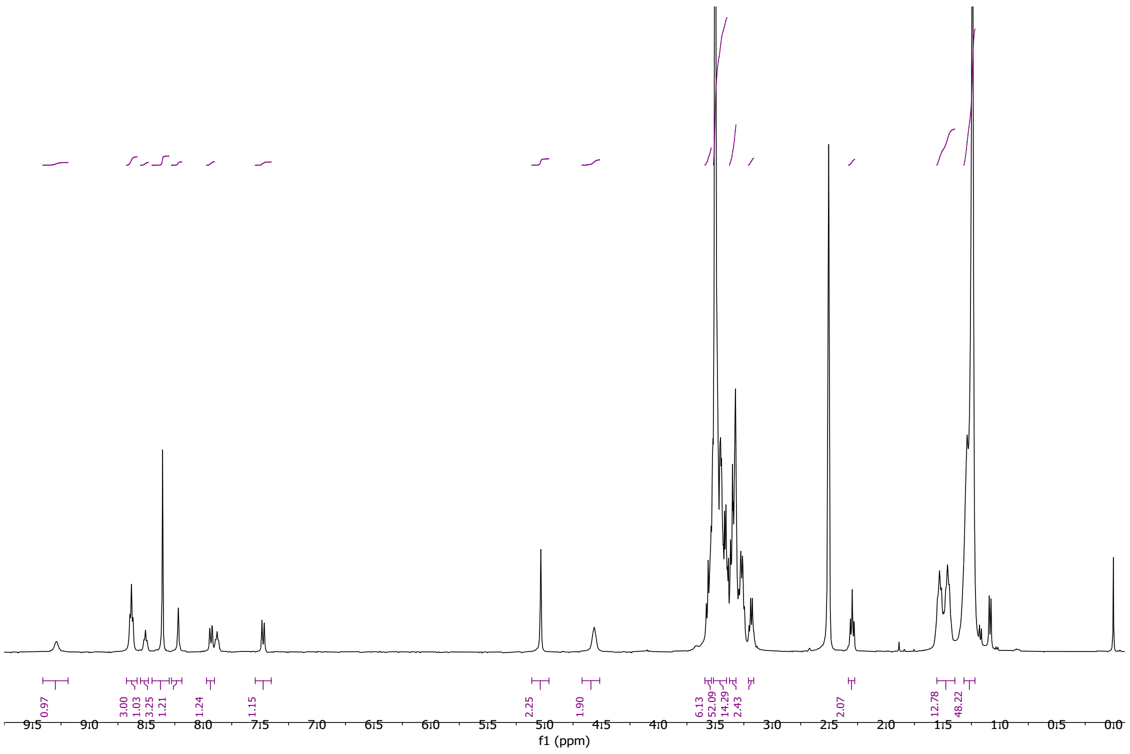

Figure S10. ^{1}H-NMR spectrum of **BTA-Ba1** in DMSO-d_6.

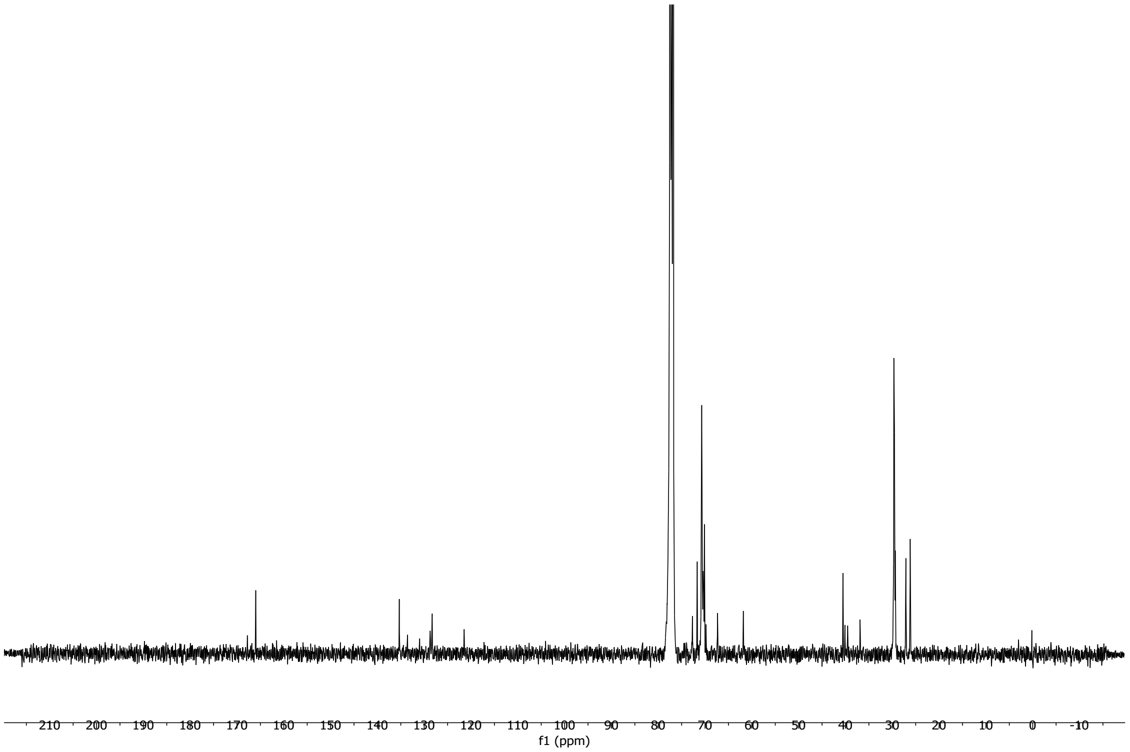

Figure S10. ^{13}C-NMR spectrum of **BTA-Ba1** in $CDCl_3$.

Synthesis of *N*[1]-(1-(1-hydroxy-1,3-dihydrobenzo[*c*][1,2]oxaborol-6-yl)-1-oxo-5,8,11,14-tetraoxa-2-azahexacosan-26-yl)-*N*[3],*N*[5]-bis(1-hydroxy-3,6,9,12-tetraoxatetracosan-24-yl)benzene-1,3,5-tricarboxamide (BTA-Ba2)

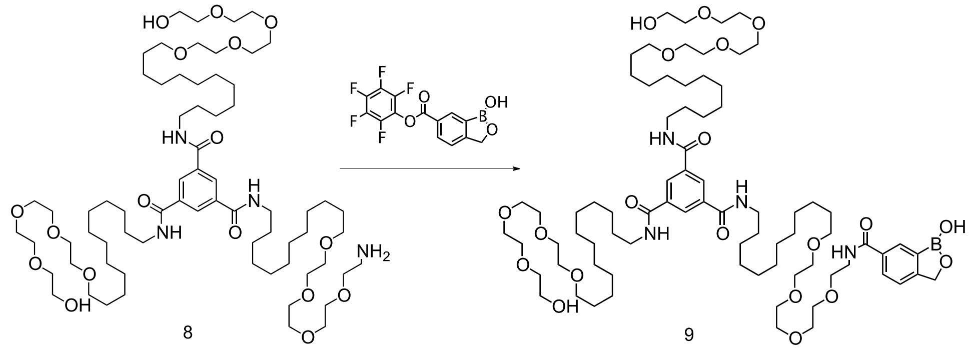

N[1]-(1-amino-3,6,9,12-tetraoxatetracosan-24-yl)-*N*[3],*N*[5]-bis(1-hydroxy-3,6,9,12-tetraoxatetracosan-24-yl)benzene-1,3,5-tricarboxamide ($BTA(OH)_2NH_2$) (**8**)[2] (149 mg; 0.11588 mmol), 96.5 mg methoxyethanol (1.27 mmol), and triethylamine (110 µL) were dissolved into 5 mL DCM. PFF-Ba (44 mg; 0.1274 mmol) was added. The reaction mixture was left under stirring for 23h. Since ^{1}H-NMR sample showed incomplete reaction, triethylamine (0.20 mL) was further added. The reaction was stopped 24h later, when the ^{1}H-NMR showed complete conversion. DCM (70 mL) was added, the mixture was transferred to a separation funnel and washed with saturated aqueous ammonium chloride (2 x 40 mL), followed by water (2 x 40 mL) and brine (70 mL). After drying over sodium sulfate, the solvent was evaporated and the crude product was obtained (69 mg). The purification was performed by column chromatography (EtOAc:MeOH 98:2 v/v to remove the impurities and $CHCl_3$:MeOH 9:1 v/v to elute the product **9**) on a Biotage Isolera One column machine (Grace 4g prepacked silica column). Yield: 34 mg (20%).

^{1}H NMR (400 MHz, DMSO) δ = 8.63 (t, *J*=5.5 Hz, 3H, $CH_2N\underline{H}C=O$), 8.50 (s, 1H, Ar (Ba)), 8.35 (s, 3H, Ar), 8.22 (s, 1H, $CH_2N\underline{H}C=OCH_2$), 7.93 (d, *J*=8.0 Hz, 1H, Ar (Ba)), 7.48 (d, *J*=8.0 Hz, 1H, Ar (Ba)), 5.03 (s, 2H, $C\underline{H_2}$-O-B), 4.58 (t, *J*=5.5 Hz, 2H, OH), 3.57 – 3.40 (m, 60H, $C=ONHCH_2C\underline{H_2}O$, $C\underline{H_2}OH$, O-$(C\underline{H_2})_2$-O), 3.28 – 3,26 (m, 2H, $C\underline{H_2}NHC=O$), 1.56 – 1.41 (m, 12H, $CH_2C\underline{H_2}CH_2O$, $C\underline{H_2}CH_2NHC=O$), 1.32 – 1.22 (m, 48H, aliphatic).
^{13}C NMR (100 MHz, $CDCl_3$) δ = 167.77, 166.14, 157.12, 135.22, 133.24, 130.19, 129.59, 128.36, 121.20, 77.39, 77.07, 76.75, 72.63, 71.60, 71.55, 70.77-70.12, 69.99, 69.88, 61.63, 40.40, 39.91, 29.52-29.14, 27.00, 26.86, 26.02, 25.90.
MS (MALDI-TOF) calcd. for $C_{77}H_{135}BN_4O_{20}$: 1469.97 $[M + Na]^+$,found 1469.97

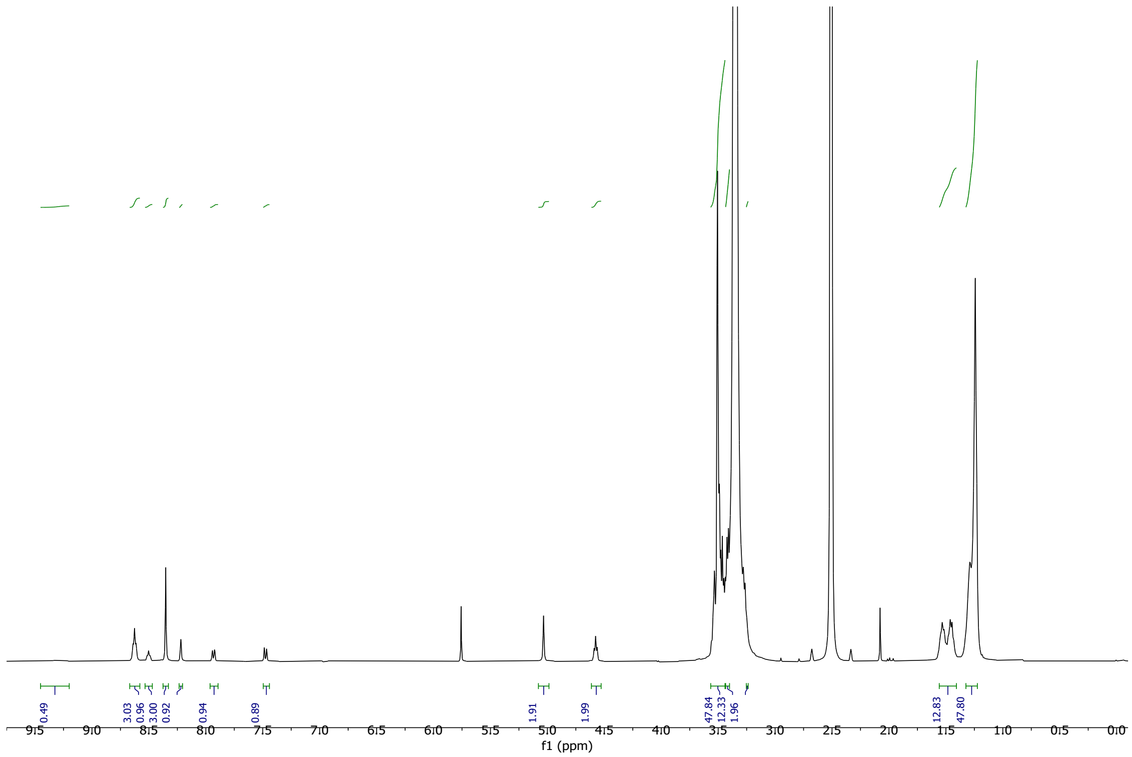

Figure S11. ^{1}H-NMR spectrum of **BTA-Ba2** in DMSO-d_6.

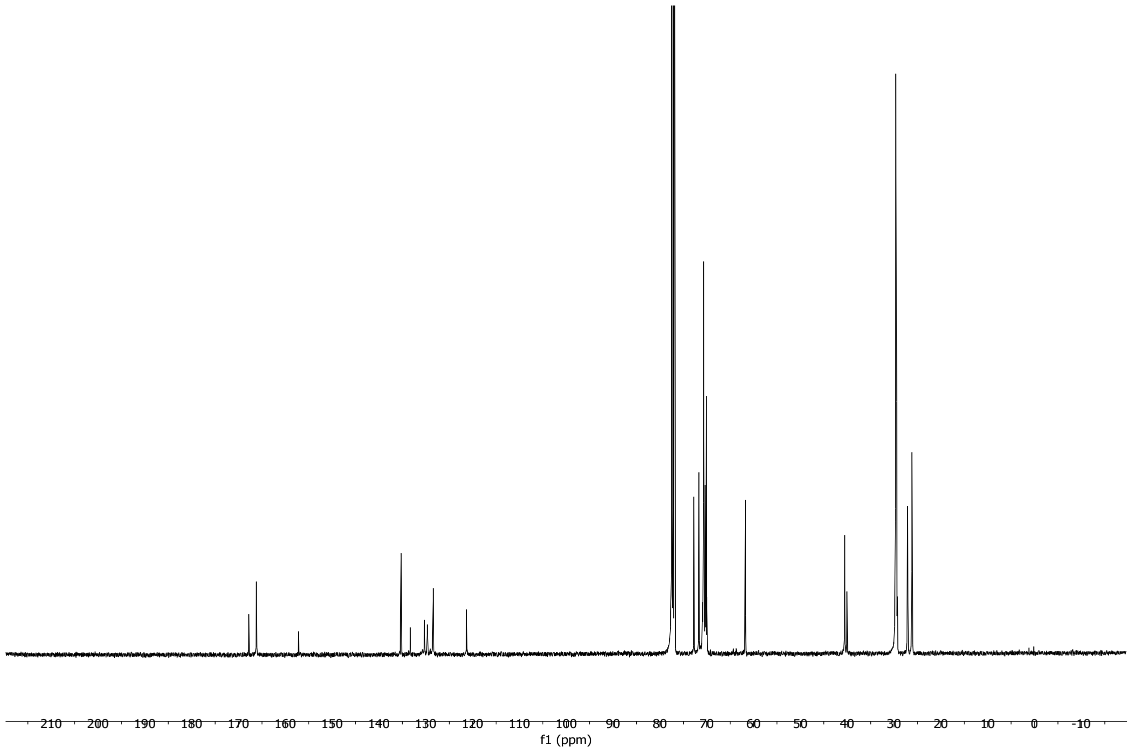

Figure S12. ^{13}C-NMR spectrum of **BTA-Ba2** in $CDCl_3$.

Synthesis of *N*^{1},*N*^{3},*N*^{5}-tris(1-(1-hydroxy-1,3-dihydrobenzo[*c*][1,2]oxaborol-6-yl)-1-oxo-5,8,11,14-tetraoxa-2-azahexacosan-26-yl)benzene-1,3,5-tricarboxamide (BTA-Ba3)

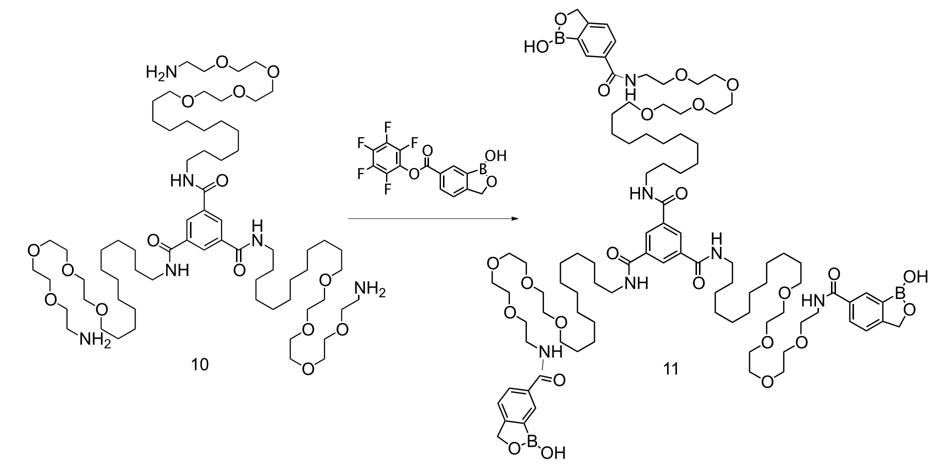

N[1],*N*[3],*N*[5]-tris(1-amino-3,6,9,12-tetraoxatetracosan-24-yl)benzene-1,3,5-tricarboxamide (BTA(NH_2)$_3$) (**10**)[4] (228 mg; 0.17732 mmol) was dissolved in dry DCM (15 mL) and triethylamine (0.22 mL) was added, followed by PFF-Ba (185 mg; 0.54 mmol). After 46h, more PFF-Ba (29 mg; 0.084 mmol) was added. The reaction was left under stirring for 6h, then *Argopore*-NH_2-LL resin (560 mg; approx. 0.157 mmol amine) was added and the reaction was stirred for other 17h. DCM (25 mL) was then added and 10 minutes later the mixture was filtered. The solid was rinsed with DCM (50 mL) and the combined organic phases were transferred in a separation funnel. The extraction was performed with saturated aqueous ammonium chloride (2 x 20 mL), followed by water (2 x 20 mL) and brine (30 mL). The organic phase was then dried with sodium sulfate, filtered and evaporated to obtain 285 mg of crude product. The purification was performed by column chromatography (gradient from $CHCl_3$ to $CHCl_3$:MeOH 99:1 v/v) on a Biotage Isolera One column machine (Biotage 10 g KP-SIL prepacked silica column). The obtained product **11** (193 mg) was dissolved in MeOH and dialysed (MWCO: 100-500 Da). Yield: 128 mg (41%).

^{1}H NMR (400 MHz, DMSO) δ = 9.28 (s, 3H), 8.62 (s, 3H, $CH_2N\underline{H}C=O$), 8.49 (s, 3H, Ar (Ba)), 8.35 (s, 3H, Ar), 8.22 (s, 3H, $CH_2N\underline{H}C=OCH_2$), 7.93 (d, *J*=8.0 Hz, 3H, Ar (Ba)), 7.47 (d, *J*=7.9 Hz, 3H, Ar (Ba)), 5.03 (s, 6H, $C\underline{H_2}$-O-B), 3.54 – 3.45 (m, 48H, O-(C$\underline{H}$2)2-O), 3.43 – 3.41 (m, 12H, CH2C$\underline{H}$2NHC=O, CH2CH2C$\underline{H}$2O), 3.28 – 3,25 (m, 6H, $C\underline{H_2}NHC=O$), 1.53 – 1.42 (m, 12H, $CH_2C\underline{H}_2CH_2O$, $C\underline{H}_2CH_2NHC=O$), 1.29 – 1.21 (m, 48H, aliphatic).
^{13}C NMR (101 MHz, $CDCl_3$) δ = 168.03, 166.35, 157.14, 135.24, 133.06, 130.87, 130.24, 129.75, 128.54, 121.10, 77.41, 77.30, 77.10, 76.78, 71.55, 70.75-70.12, 69.97, 69.82, 69.76, 40.43, 40.05, 29.52-29.21, 27.01, 26.93, 25.93
MS (MALDI-TOF) calcd. for $C_{93}H_{147}B_3N_6O_{24}$: 1788.06 [M + Na]$^+$, found 1788.07

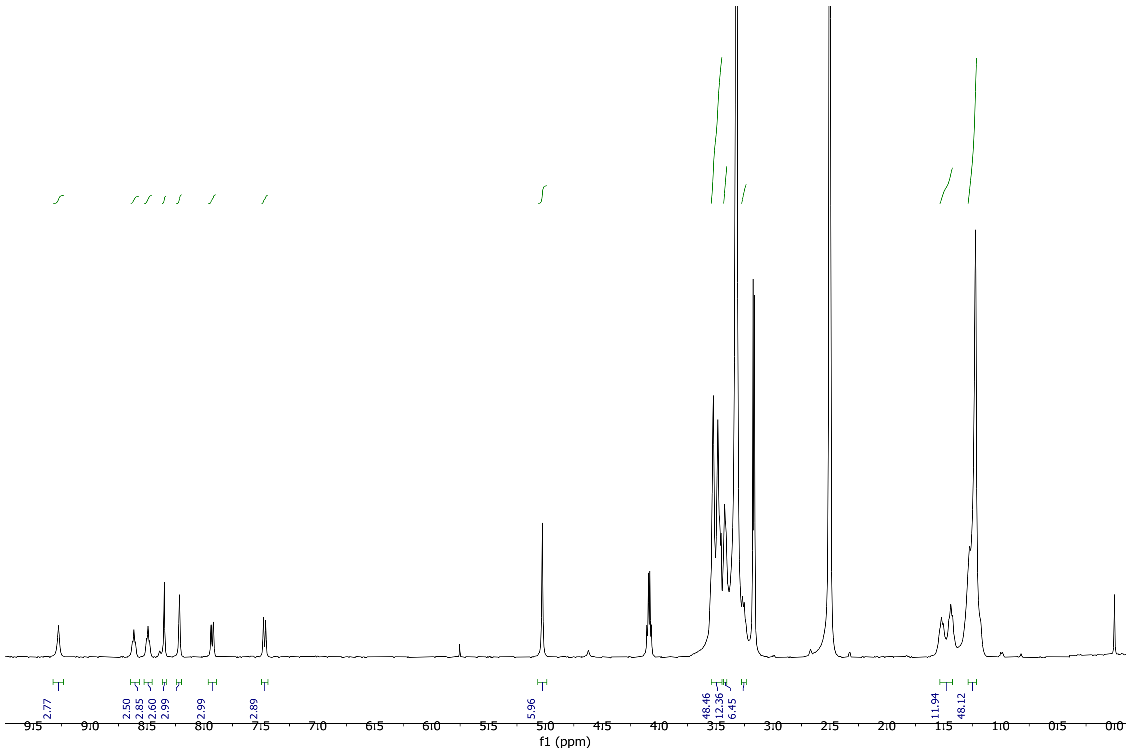

Figure S13. ^{1}H-NMR spectrum of **BTA-Ba3** in DMSO-d_6.

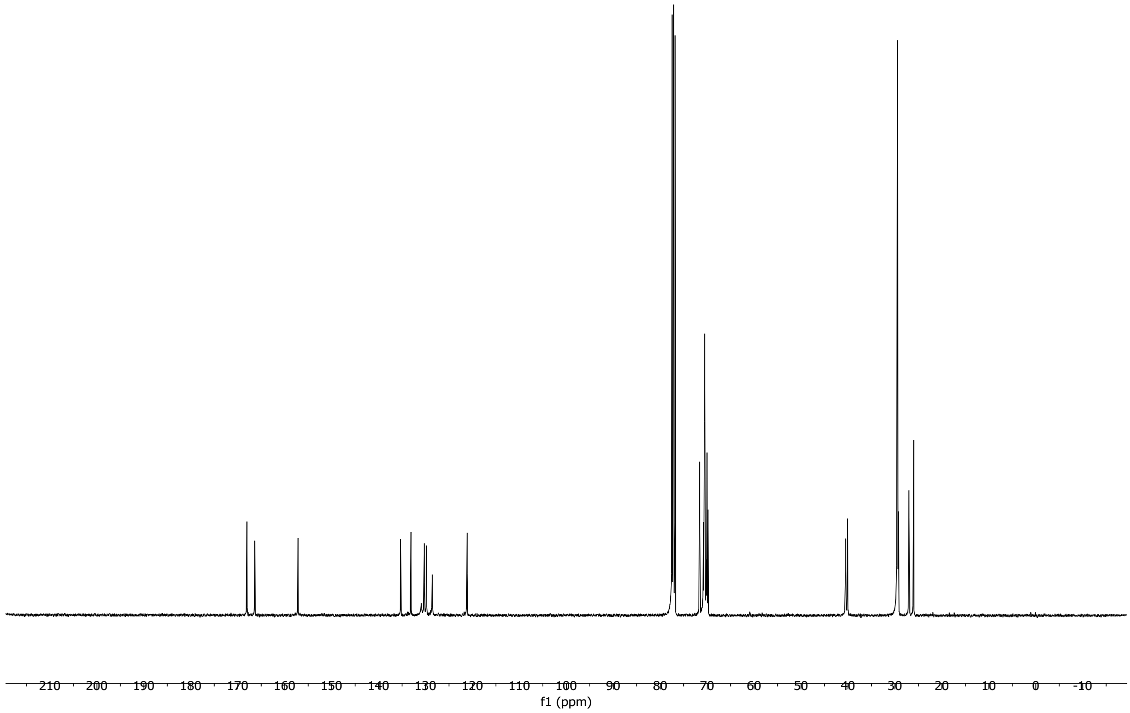

Figure S14. ^{13}C-NMR spectrum of **BTA-Ba3** in $CDCl_3$.

Synthesis of 1-hydroxy-*N*-(2-(2-(2-hydroxyethoxy)ethoxy)ethyl)-1,3-dihydrobenzo[*c*][1,2]oxaborole-6-carboxamide (OEG$_3$-Ba)

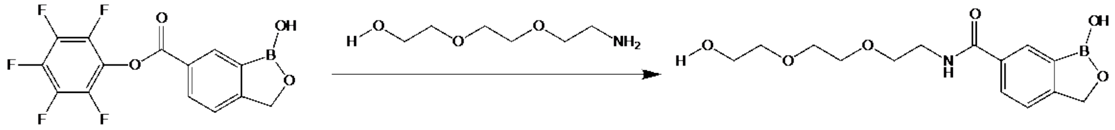

PFF-Ba (97 mg; 0.282 mmol) was stirred with 1 mL DCM. Triethylamine (0.17 mL) was added to this turbid mixture and the solution became clear within one minute. A solution of OEG_3-NH_2 (71 mg; 0.563 mmol in 2 mL DCM) was added dropwise and the mixture was stirred for 21h at room temperature, prior to evaporation of the solvent. The purification was performed by column chromatography (90:9:1 $CHCl_3$:MeOH:acetic acid) on a Biotage Isolera One column machine (KP-Sil 25 g prepacked silica column). The product was co-evaporated twice with $CHCl_3$ and dried in a high vacuum. Yield: 84 mg (96 %).

^{1}H NMR (400 MHz, DMSO) δ = 9.30 (s, 1H, B-OH), 8.51 (t, *J*=5.6 Hz, 1H, Ar (Ba)), 8.22 (d, *J*=1.8 Hz, 1H, $CH_2N\underline{H}C{=}OCH_2$), 7.93 (d, *J*=8.0 Hz, 1H, Ar (Ba)), 7.48 (d, *J*=8.0 Hz, 1H, H_{arom} (Ba)), 5.04 (s, 2H, $C\underline{H_2}$-O-B), 4.58 (s, 1H, OH), 3.56 – 3.52 (m, 6H, $OC\underline{H_2}C\underline{H_2}OH$, $C\underline{H_2}O$), 3.49 – 3.45 (m, 2H, $NHC\underline{H_2}$), 3.43 – 3.39 (m, 4H, $OC\underline{H_2}C\underline{H_2}O$).
^{13}C NMR (100 MHz, DMSO) δ = 167.10, 157.21, 130.19, 129.96, 121.71, 79.66, 72.82, 70.43, 70.20, 70.11, 69.37, 60.69, 40.63, 40.42, 40.21, 40.00, 39.79, 39.58, 39.37.
MS (LC) calcd. for $C_{14}H_{20}BNO_6$: 310.14 $[M + H]^+$,found 310.00

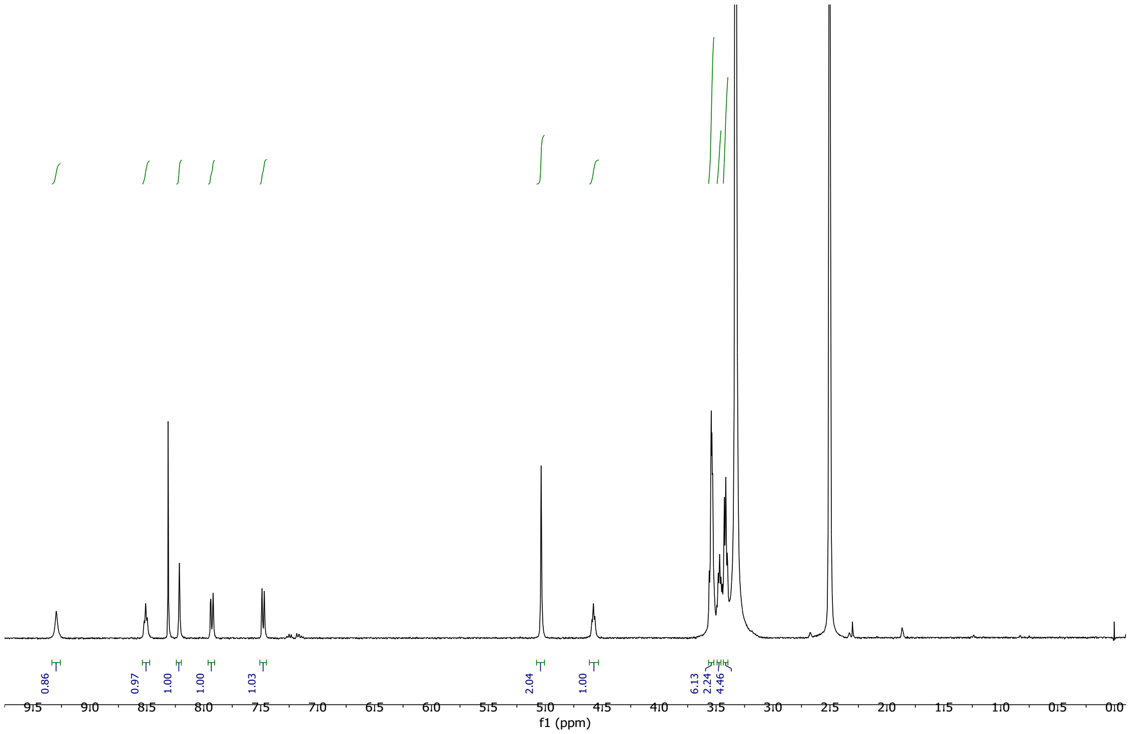

Figure S15. ^{1}H-NMR spectrum of OEG_3-Ba in DMSO-d_6.

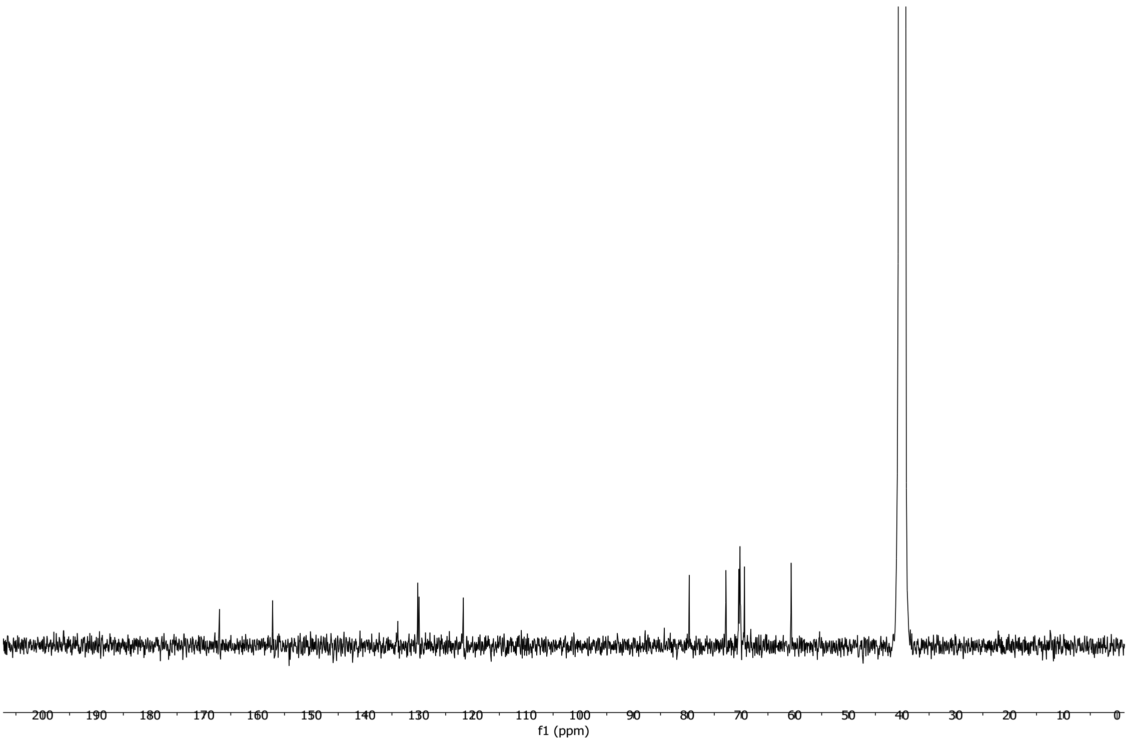

Figure S16. ^{13}C-NMR spectrum of OEG_3-Ba in DMSO-d_6.

Nuclear magnetic resonance (NMR)

^{1}H-NMR and ^{13}C-NMR spectra were recorded on a Bruker 400 MHz Ultrashield spectrometer. The specific deuterated solvent is indicated in each case.

Matrix assisted laser absorption/ionization-time of flight (MALDI-TOF)

MALDI-TOF mass spectra were recorded on a PerSeptive Biosystems Voyager DE-PRO spectrometer using α-cyano-4-hydroxycinnamic acid (CHCA) or trans-2- [3-(4-tert-butylphenyl)-2-methyl-2- propenylidene]-malononitrile (DCTB) as matrix.

Liquid chromatography mass spectrometry (LC-MS)

LC-MS was performed using a Thermo Finnigan LCQ Fleet ion trap mass spectrometer equipped with a Surveyor autosampler and a Thermo Finnigan Surveyor PDA detector. Prior to mass analysis, samples were separated on a reversed phase C18 column in acetonitrile/water/0.1% formic acid gradients using Shimadzu SCL-10A pumps.

Assembly of BTA-Ba:BTA-3OH copolymers

The assembly of BTA-Ba:BTA-3OH copolymers was performed as previously reported.[5] In particular, BTA-Ba and BTA-3OH monomers were weighted to obtain the desired molar ratio and water was added to reach the desired concentration. The mixture was then heated at 90°C under stirring for 15 minutes, vortexed for 15 seconds while still hot and left to equilibrate overnight at room temperature (RT).

UV-Vis spectroscopy

UV-Vis spectra were recorded on a Jasco V-750 UV-Vis spectrometer equipped with a temperature controller. Measurements were performed on 50 μM BTA samples in a Quartz cuvette with a path length of 1 cm at 20 °C. MilliQ water was used as reference. All

measurements were performed with a bandwidth of 1.0 nm, a scan speed of 100 nm/min and a data interval of 0.1 nm, recording from 190 nm to 350 nm. In order to follow the assembly/disassembly over temperature, heating-cooling programs were performed using a temperature ramp of 0.1 °C/min.

Static light scattering (SLS) measurements

SLS measurements were recorded on an ALV/CGS-3 MD-4 compact goniometer system equipped with a multiple tau digital real time correlator (ALV-7004) and a solid state laser (λ = 532 nm; 40 mW). Scattering intensity was detected over an angular range of 30° to 150° with steps of 10°, and averaged over at least 10 runs of 10 seconds per angle. The samples were prepared at a concentration of 500 μM. As references, MilliQ water and toluene were measured. The measurements were analysed using a Matlab script.

Cryogenic transmission electron microscopy (Cryo-TEM)

Imaging was performed on samples with a BTA concentration of 500 μM in MilliQ-water. Vitrified films were prepared in a 'Vitrobot' instrument (PC controlled vitrification robot, patent applied, Frederik et al 2002, patent licensed to FEI, Vitrobot™ Mark III) at 22°C and at a relative humidity of 100%. In the preparation chamber of the 'Vitrobot', 3 μL samples were applied on Lacey grids (LC200-Cu, Electron Microscopy Sciences), which were surface plasma treated just prior to use (Cressington 208 carbon coater operating at 5 mA for 40 s). Excess sample was removed by blotting using filter paper for 4 s at –3 mm, and the thin film thus formed was plunged (acceleration about 3 g) into liquid ethane just above its freezing point. Vitrified films were transferred into the vacuum of a CryoTITAN equipped with a field emission gun that was operated at 300 kV, a post-column Gatan energy filter, and a 2048 x 2048 Gatan CCD camera. Virtrified films were observed in the CryoTITAN microscope at temperatures below -170 °C. Micrographs were taken at low dose conditions, starting at a magnification of 6500 with a defocus setting of -40 μm, and at a magnification of 24000 with a defocus setting of -10 μm.

Total internal reflection fluorescence (TIRF) microscopy

TIRF images were acquired with a Nikon N-STORM system. Cy3 was excited using a 561 nm laser. Fluorescence was collected by means of a Nikon×100, 1.4NA oil immersion objective and passed through a quad-band pass dichroic filter (97335 Nikon). Images were recorded with an EMCCD camera (ixon3, Andor, pixel size 0.17 μm). BTA fibers were prepared at a total BTA concentration of 50 μM with 5mol% of Cy3-BTA and different % of BTA-Ba. The samples were first diluted to 25 μM total BTA with 2xPBS and then further diluted to 2.5 μM prior to the measurement. In order to image the fibers, 35 μL of a 1 mg/mL poly-L-lysine (PLL) solution were first flown in a chamber between a glass microscope coverslip (Menzel-Gläser, no. 1, 21 × 26 mm) and a glass slide, which were separated by double-sided tape. After 5 minutes, MilliQ-water was injected into the chamber to remove the excess of PLL, followed by 35 μL of the sample.

Assembly of BTA-Ba:BTA-3OH:BTA-Cy3 copolymers

All the copolymers used for TIRF microscopy were labeled with 5mol% of Cy3-BTA (with respect to the total amount of BTA). The amount of Cy3-BTA was always kept constant and different samples were prepared choosing a specific molar ratio of BTA-Ba and BTA-3OH. First of all, BTA-Ba and BTA-3OH monomers were weighted in a vial and water was added. Then, the vial was heated at 90°C under stirring for 15 minutes and vortexed for 10 seconds while still hot. After 5 minutes, a MeOH solution containing 5mol% of Cy3-BTA (with respect to the total amount of BTA) was added and the solution was vortexed again for 10 seconds. This solution was finally heated at 45°C without stirring for other 15 minutes, before equilibration overnight in the dark at RT.

Alizarin Red S (ARS) assay

In order to assess the interaction between BTA-Ba1:BTA-3OH fibers and carbohydrates, the ARS assay was performed.[6] In a 96-well plate, 50 µL of ARS 40 nM in 2xPBS was added to 50 µL of BTA-Ba1:BTA-3OH (1 mM in MilliQ water). The fluorescence spectrum was measured in the range from 520 nm to 800 nm, exciting at 470 nm, using a plate reader TECAN Spark 10M. By titrating 100 µL sialic acid (from 1 nM to 250 mM in PBS) into the ARS/fiber solution, the displacement of ARS with the carbohydrate was monitored by following the fluorescence quenching. In order to take into account the dilution effect upon titration, the spectrum of the control (50 µL BTA-Ba1 + 50 µL ARS + 100 µL PBS) was reported as 0 mM sialic acid in all the graphs. The measurements were performed in triplicates and the average spectrum for each sialic concentration was reported.

Fluorescence anisotropy

In a 384-micro well plate, 5 µM ARS solution in 2xPBS was reacted with increasing amount of BTA-Ba1:BTA-3OH 1:99 fibers and OEG_3-Ba in MilliQ water. Fluorescence anisotropy was measured in triplicates using a plate reader TECAN Spark 10M, exciting at 470 nm and recording fluorescence polarization at 550nm.

Human red blood cells (hRBCs) thawing protocol

12% NaCl, 1.6% NaCl and 0.9% NaCl + 0.2% glucose solutions in MilliQ-water were prepared and warmed up at 37 °C. 2 mL hRBCs were transferred directly from -80°C to 37°C and thawed for 2 minutes. Then the hRBCs were transferred into a 50 mL Falcon tube and 0.2 mL of 12% NaCl were added, drop by drop with gentle shaking. 11 mL of 1.6% NaCl were further slowly added always drop by drop with side-to-side gentle shaking. The suspension was then centrifuged at 400g for 5 minutes with low deceleration. The supernatant was then discarded and 9 mL of 0.9% NaCL:0.2% glucose was added dropwise before centrifuging once more at 400g for 3 minutes always using low brake. Finally, the supernatant was removed and the pellet was suspended in PBS.

Interaction study between hRBCs and BTA-Ba1:BTA-3OH:BTA-Cy3 copolymers

The interaction between hRBCs and BTA-Ba1:BTA-3OH:BTA-Cy3 copolymers was studied by means of TIRF microscopy. Prior to imaging, the fibers were incubated with hRBC for 1h. In particular, 2 µL of the hRBC thawed pellet were transferred in an eppendorf and diluted with 100 µL of PBS (suspension A). The 50 µM BTA-Ba1:BTA-3OH:BTA-Cy3 fibers solution in MilliQ-water was diluted to 12.5 µM in PBS (solution B). The solution B was then added to the suspension A in 1:1 volume ratio and the mixture was left on the shaking plate at RT. After 1h, the suspension was centrifuged at 300g for 5 minutes to remove the unbound fibers and the supernatant was replaced by 150 µL of PBS.

35 µL of PLL solution were then injected into the microscopy chamber and washed with MIlliQ water after 5 minutes. The suspension hRBCs/BTA fibers was then flown into the chamber and the glass slide was flipped upside down to allow cell adhesion. The system was left in this position for 10 minutes, before imaging using 200 ms exposure and 8% of laser intensity.

2. Supplementary Figures and Movies

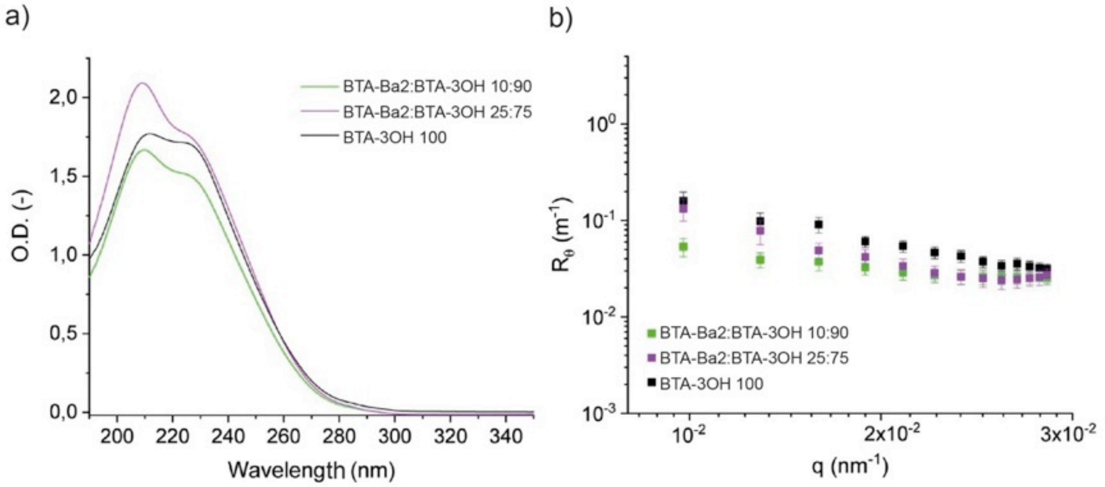

Figure S17. UV-Vis spectra (a) and SLS plot (b) BTA-Ba2:BTA-3OH copolymers compared with those of BTA-3OH homopolymers.

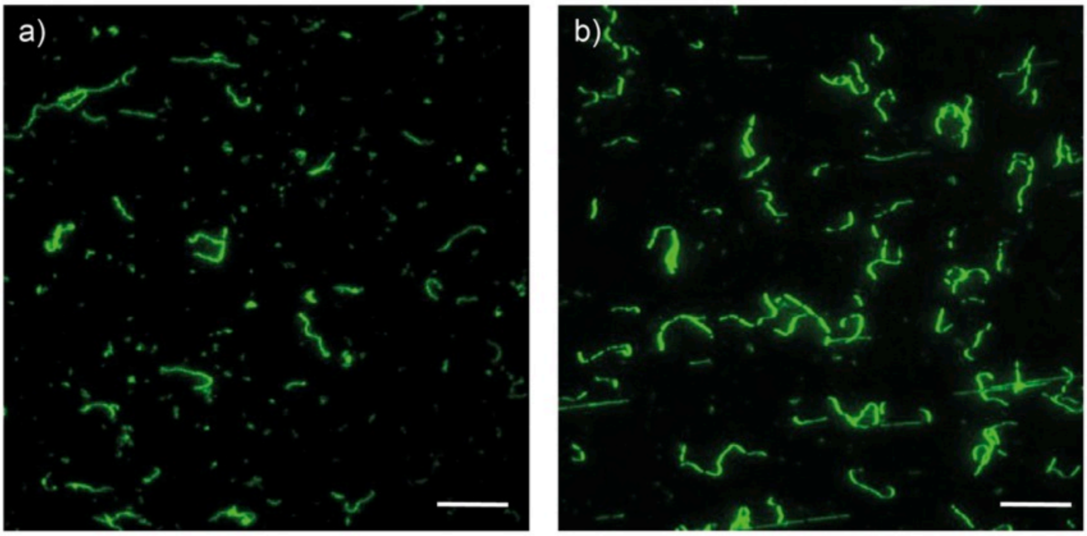

Figure S18. TIRF BTA-Ba2:BTA-3OH 10:90 (a) and 25:75 (b). Scale bar 10 μm

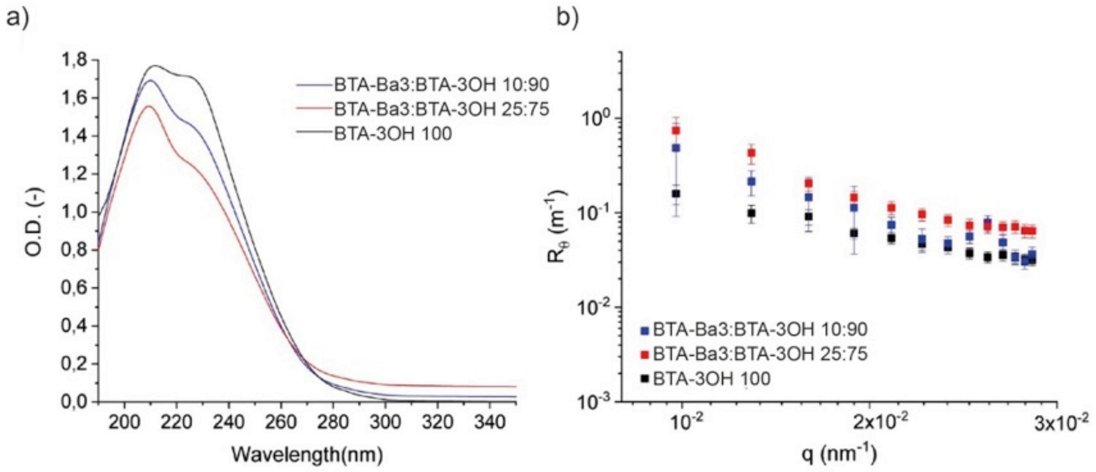

Figure S19. UV-Vis spectra (a) and SLS plot (b) BTA-Ba3:BTA-3OH copolymers compared with those of BTA-3OH homopolymers.

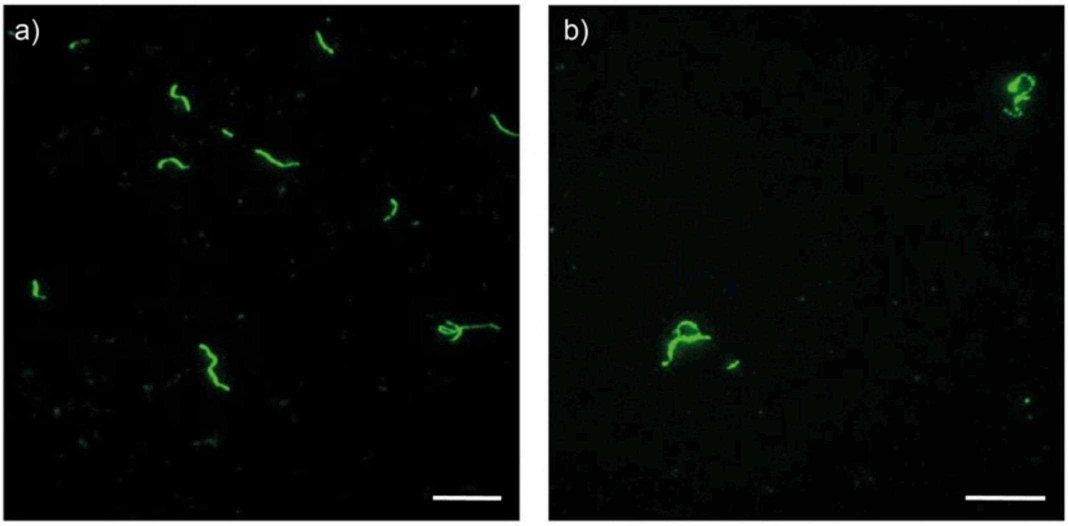

Figure S20. TIRF BTA-Ba3:BTA-3OH 10:90 (a) and 25:75 (b). Scale bar 10 μm

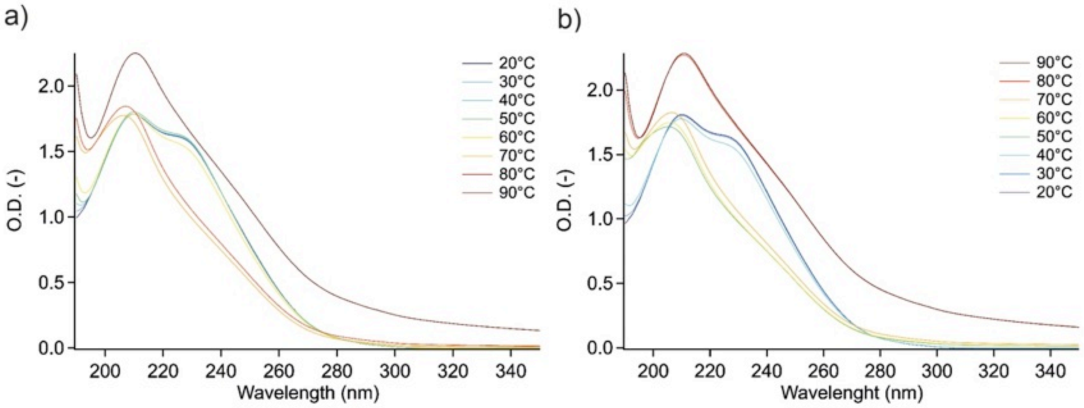

Figure S21. Temperature-dependent UV-Vis spectra of BTA-Ba1:BTA-3OH 10:90 during heating (a) and cooling (b).

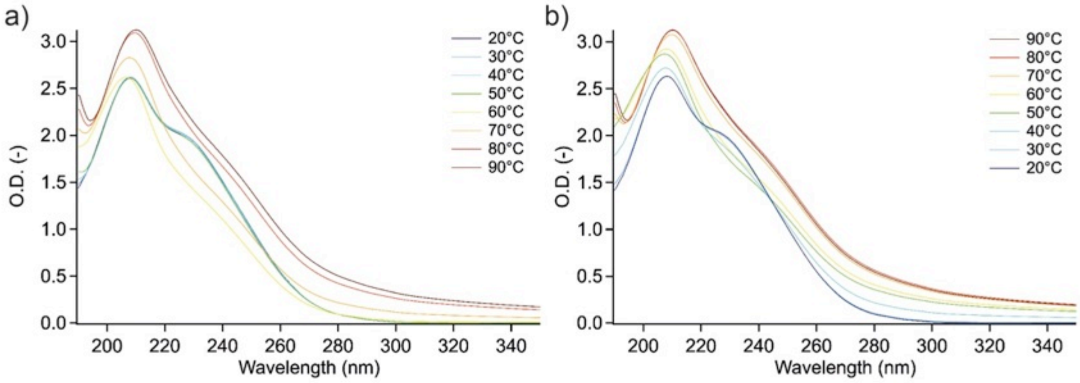

Figure S22. Temperature-dependent UV-Vis spectra of BTA-Ba1:BTA-3OH 25:75 during heating (a) and cooling (b).

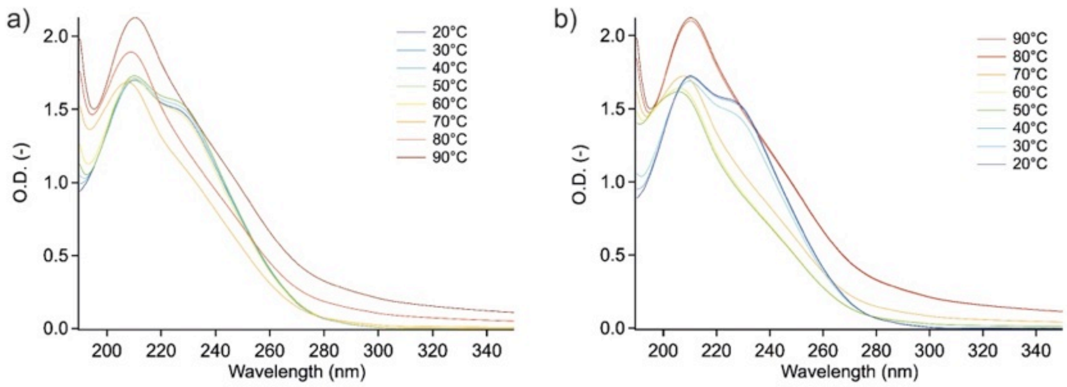

Figure S23. Temperature-dependent UV-Vis spectra of BTA-Ba2:BTA-3OH 10:90 during heating (a) and cooling (b).

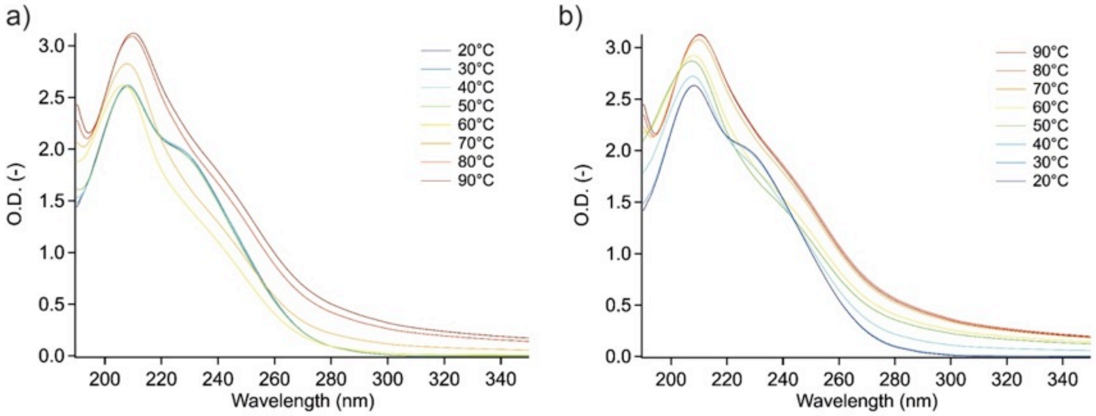

Figure S24. Temperature-dependent UV-Vis spectra of BTA-Ba2:BTA-3OH 25:75 during heating (a) and cooling (b).

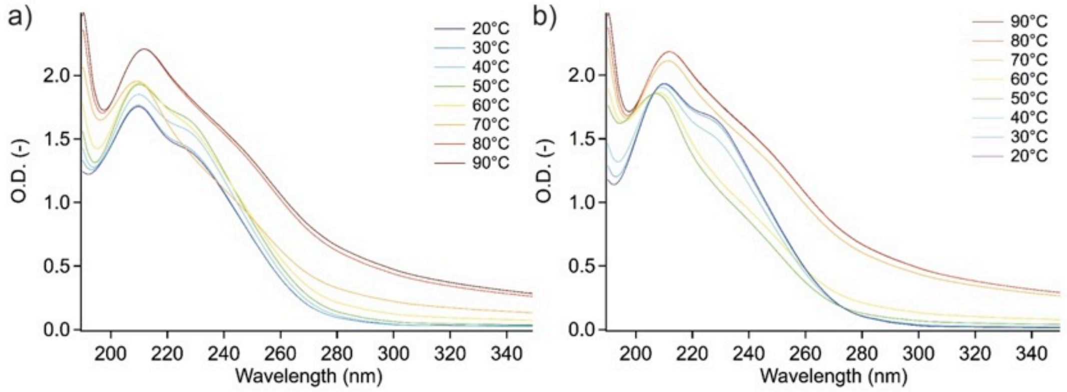

Figure S25. Temperature-dependent UV-Vis spectra of BTA-Ba3:BTA-3OH 10:90 during heating (a) and cooling (b).

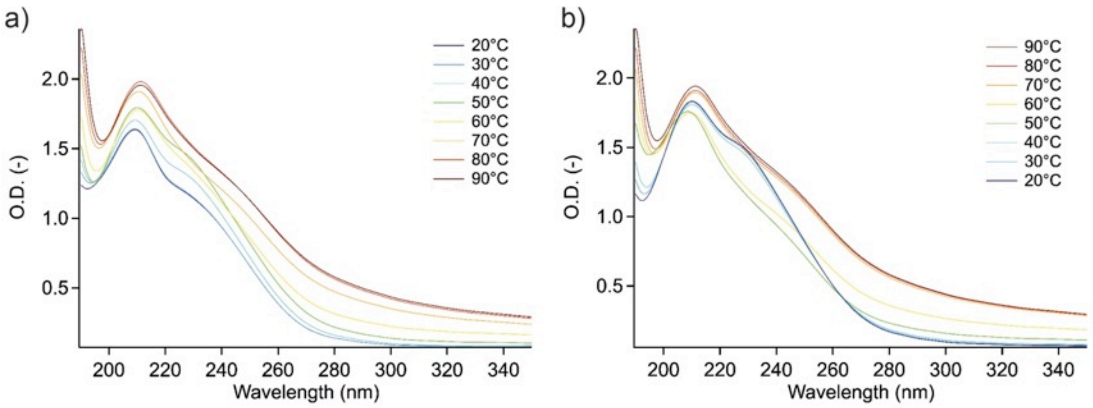

Figure S26. Temperature-dependent UV-Vis spectra of BTA-Ba3:BTA-3OH 25:75 during heating (a) and cooling (b).

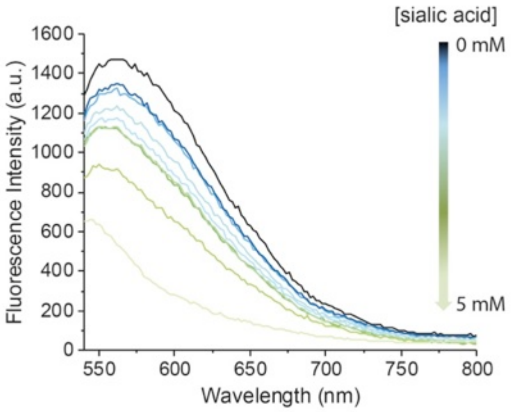

Figure S27. ARS assay on BTA-Ba1:BTA-3OH 5:95 upon titration with sialic acid.

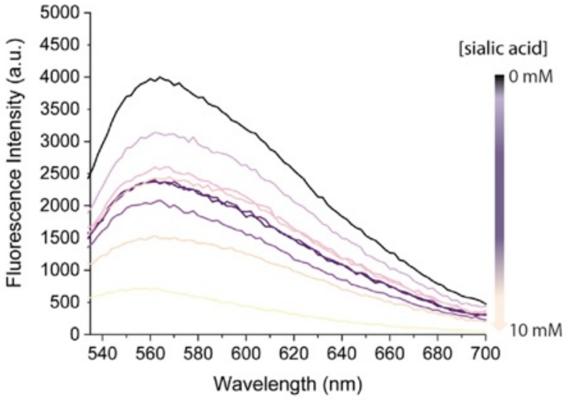

Figure S28. ARS assay on BTA-Ba1:BTA-3OH 10:90 upon titration with sialic acid.

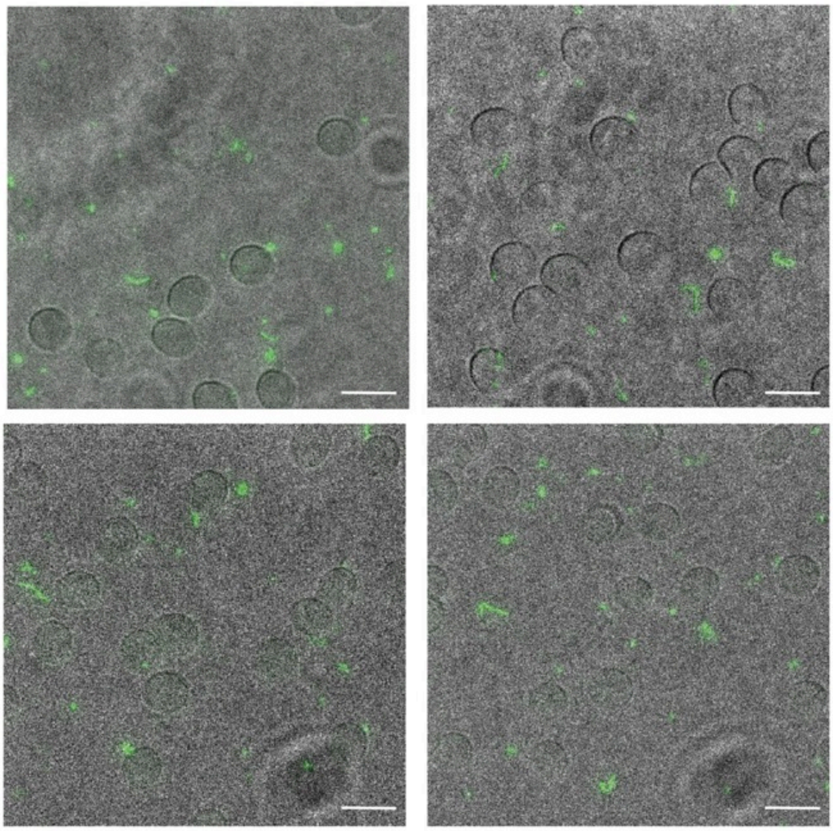

Figure S29. Superimposition of TIRF and transmission images of different samples of hRBCs incubated for 1h with BTA-3OH 100%, showing no interaction. Scale bar 10 μm

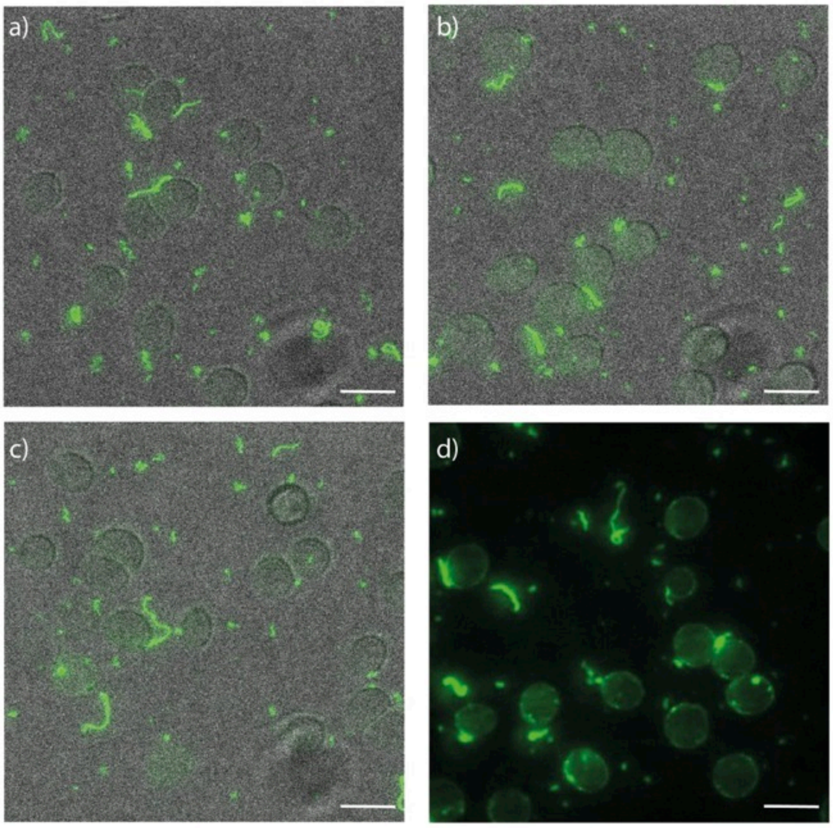

Figure S30. Superimposition of TIRF and transmission images (a, b, c) and TIRF image (d) of hRBCs incubated for 1h with BTA-Ba1:BTA-3OH 0.1:99.9. Scale bar 10 μm.

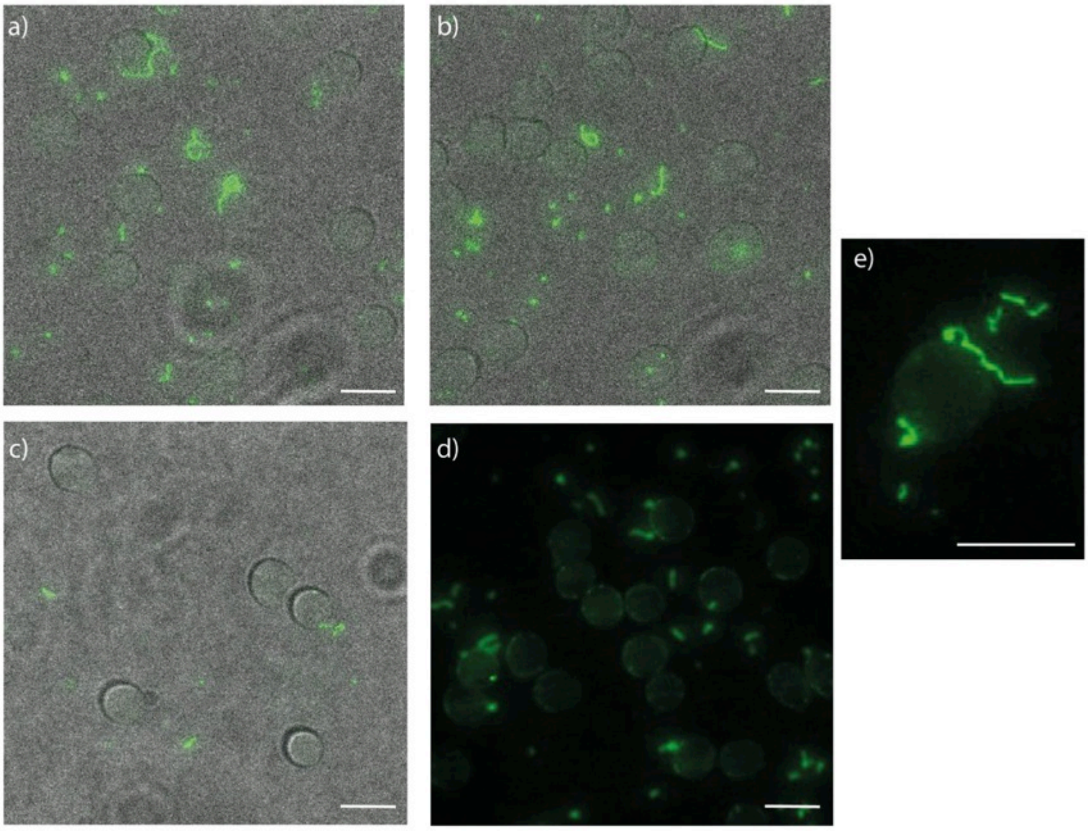

Figure S31. Superimposition of TIRF and transmission images (a, b, c) and TIRF images (d, e) of hRBCs incubated for 1h with BTA-Ba1:BTA-3OH 0.5:99.5. Scale bar 10 µm.

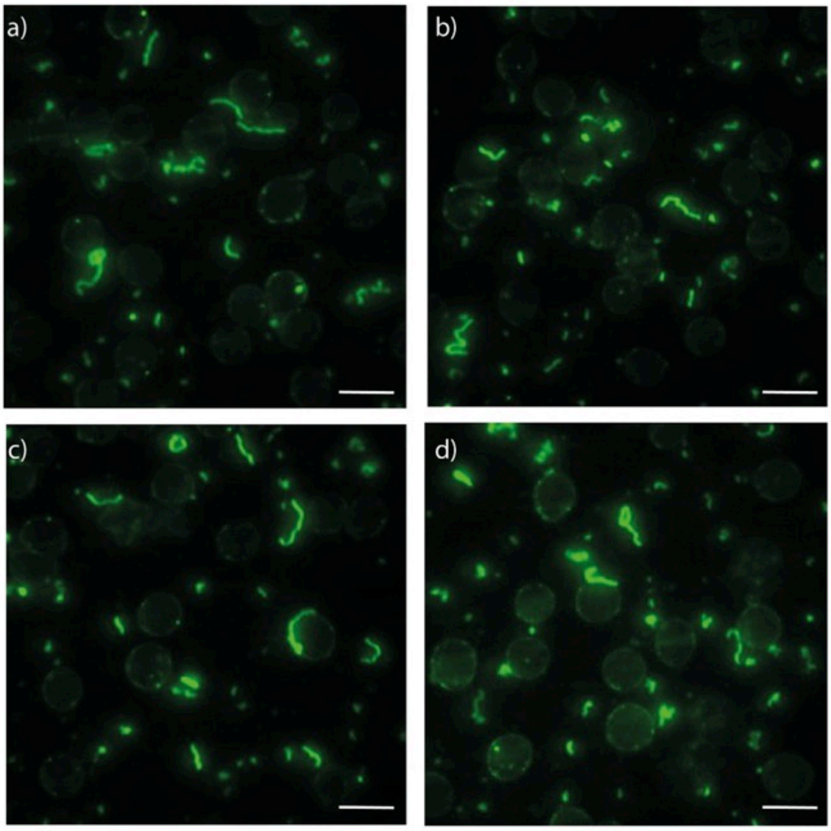

Figure S32. TIRF images of hRBCs incubated for 1h with BTA-Ba1:BTA-3OH 1:99. Scale bar 10 µm.

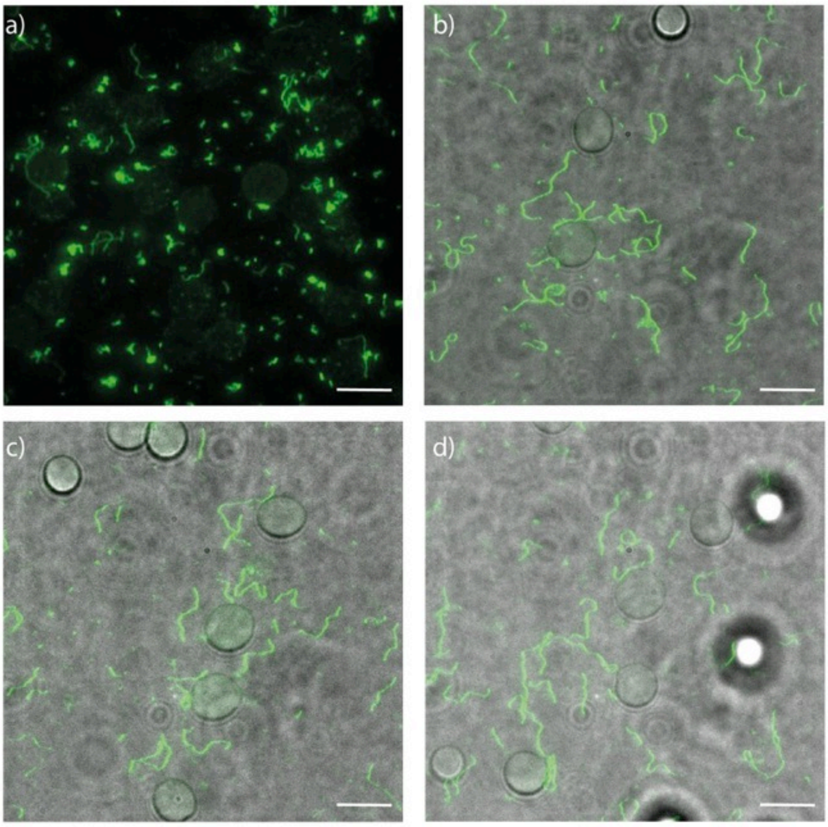

Figure S33. TIRF image (a) and superimposition of TIRF and transmission images (b, c, d) of hRBCs incubated for 1h with BTA-Ba1:BTA-3OH 5:95. Scale bar 10 µm

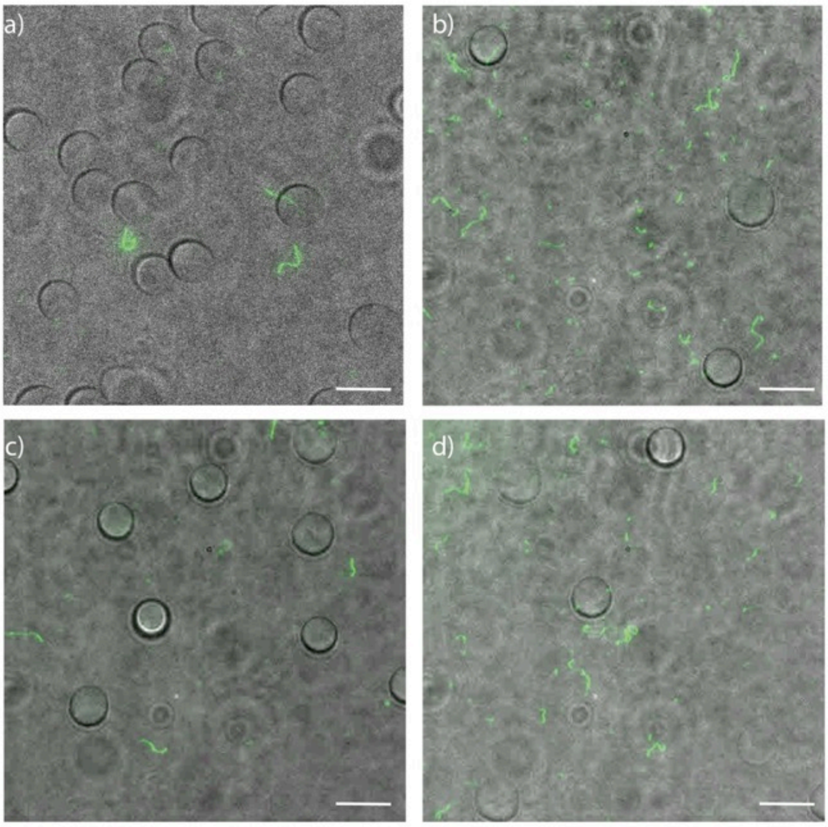

Figure S34. Superimposition of TIRF and transmission images of hRBCs incubated for 1h with BTA-Ba1:BTA-3OH 10:90. Scale bar 10 µm.

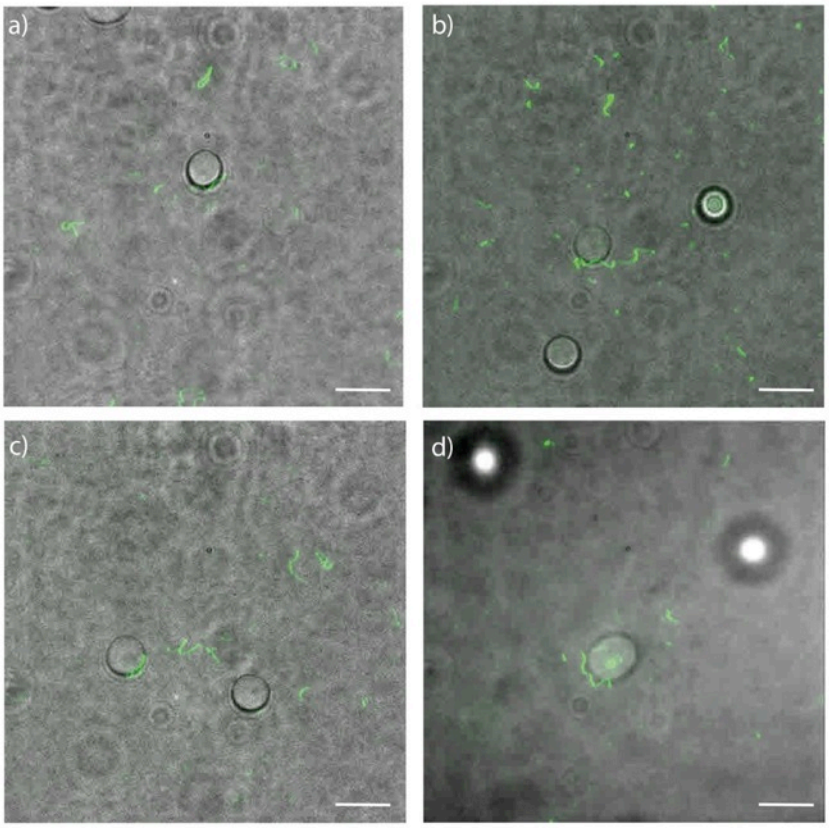

Figure S35. Superimposition of TIRF and transmission images of hRBCs incubated for 1h with BTA-Ba1:BTA-3OH 1:99 and exposed to sialic acid (10x in concentration with respect to the total amount of Ba). Scale bar 10 µm.

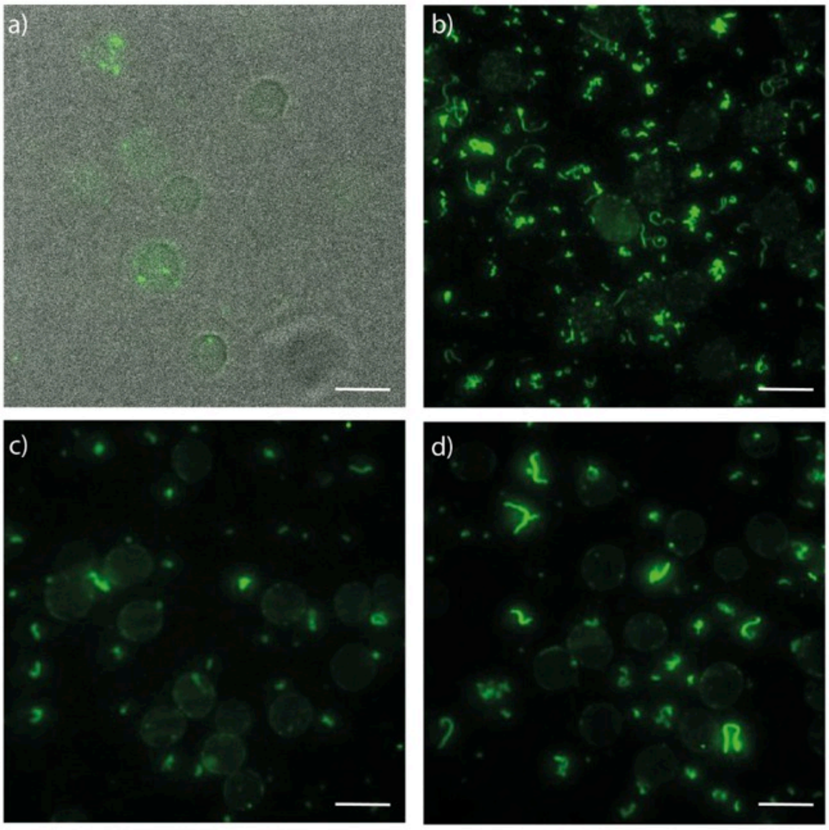

Figure S36. Superimposition of TIRF and transmission image (a) and TIRF images (b, c, d) of hRBCs incubated for 1h with BTA-Ba1:BTA-3OH 1:99 and exposed to sialic acid (100x in concentration with respect to the total amount of Ba). Scale bar 10 μm.

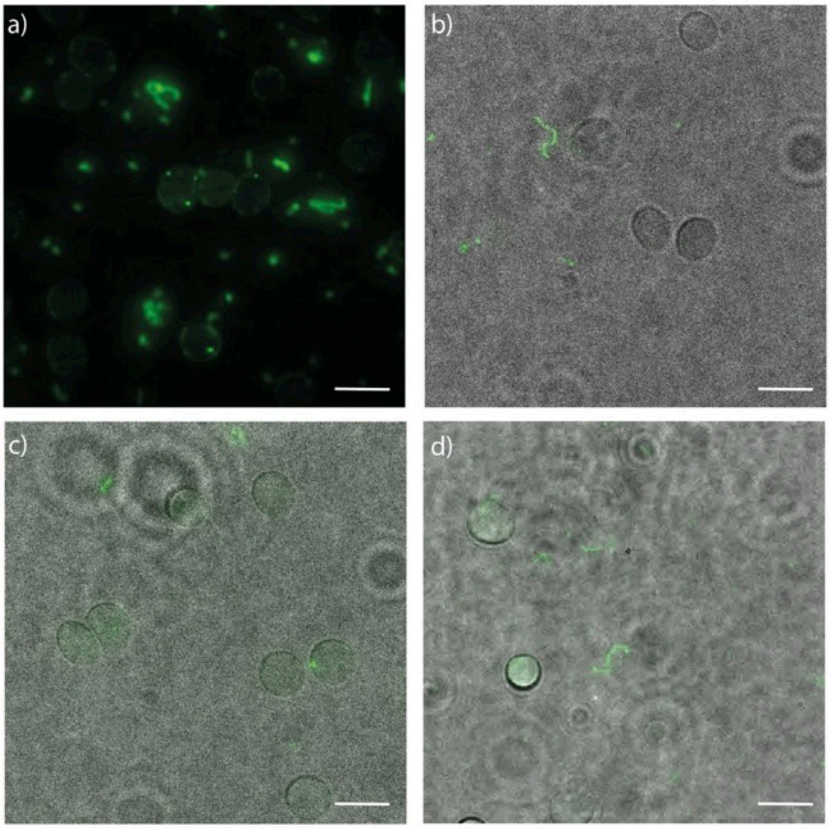

Figure S37. TIRF image (a) and superimposition of TIRF and transmission images (b, c, d) of hRBCs incubated for 1h with BTA-Ba1:BTA-3OH 1:99 and exposed to sialic acid (1000x in concentration with respect to the total amount of Ba). Scale bar 10 μm.

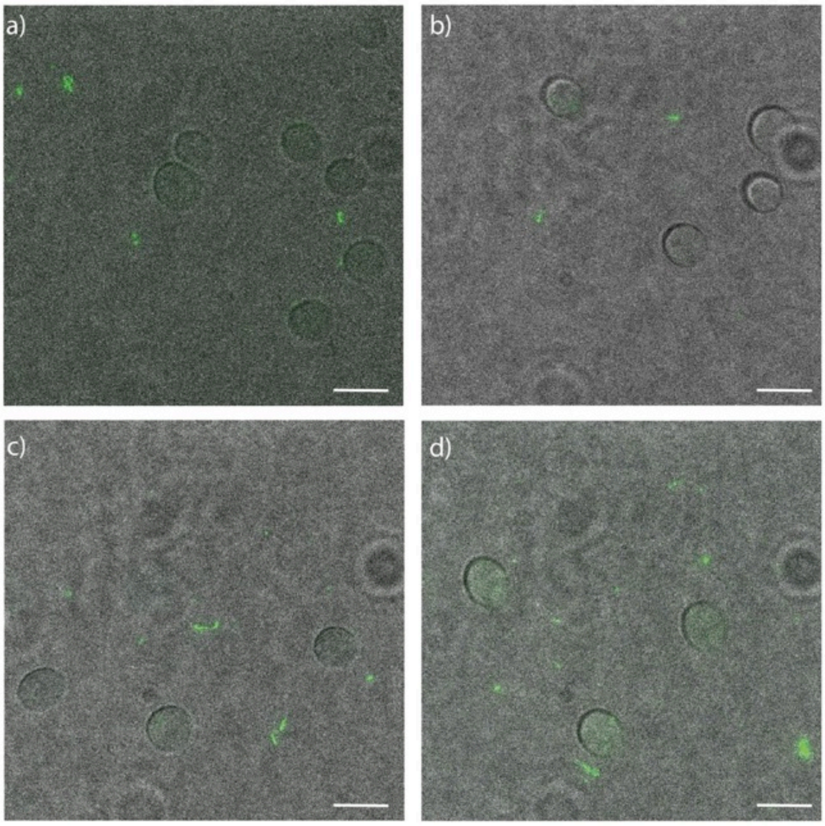

Figure S38. Superimposition of TIRF and transmission images of hRBCs incubated for 1h with BTA-Ba1:BTA-3OH 1:99 and exposed to sialic acid (10000x in concentration with respect to the total amount of Ba). Scale bar 10 µm.

Movie S1. TIRF real-time imaging of BTA-Ba1:BTA-3OH 0.5:9.5 anchored on one hRBC with one extremity and moving in solution with the other extremity.

Movie S2. TIRF real-time imaging of BTA-Ba1:BTA-3OH 1:9 anchored on one hRBC with one extremity and moving in solution with the other extremity.

3. References

1. Leenders, C. M. A.; Albertazzi, L.; Mes, T.; Koenigs, M. M. E.; Palmans, A. R. A.; Meijer, E. W. *Chem. Commun.* **2013**, *49* (19), 1963.
2. Albertazzi, L.; van der Zwaag, D.; Leenders, C. M. A.; Fitzner, R.; van der Hofstad, R. W.; Meijer, E. W. *Science* **2014**, *344* (6183), 491.
3. Matsumoto, N. M.; Lafleur, R. P. M.; Lou, X.; Shih, K-C.; Wijnands, S. P. W.; Guibert, C.; van Rosendaal J. W. A. M.; Voets, I. K.; Palmans, A. R. A., Lin, Y.; Meijer, E. W. *J. Am. Chem. Soc.* **2018**, *140*, 41, 13308-13316.
4. Leenders, C. M. A.; Baker, M. B.; Pijpers, I. A. B.; Lafleur, R. P. M.; Albertazzi, L.; Palmans, A. R. A.; Meijer, E. W. *Soft Matter*, **2016**,12, 2887-2893.
5. Lou X., Lafleur R. P. M., Leenders C. M. A., Schoenmakers S. M. C., Matsumoto N. M., Baker M. B.,. van Dongen J. L. J, Palmans A. R. A. and Meijer E. W., *Nat. Commun*. **2017**, *8*, 15420.
6. Brooks, W. L. A.; Deng, C. C.; Sumerlin B. S. *ACS Omega* **2018**, *3*, 12, 17863.
